# Quinoxaline-based cyanoacrylate hybrids as multi-target anti-proliferative and anti-inflammatory agents with potent activity against hepatocellular carcinoma: induction of apoptosis and modulation of oxidative stress signaling

**DOI:** 10.1039/d6ra03332f

**Published:** 2026-08-21

**Authors:** Samar Zuhair Alshawwa, Ghazi A. Bamagous, Hatem Adel M. Sembawa, Lara Mubarak A Almutlaqah, Aljawharah Mohammad Alkhodair, Amal Abdullah Alrashidi, Essa M. Saied

**Affiliations:** a Department of Pharmaceutical Sciences, College of Pharmacy, Princess Nourah Bint Abdulrahman University P. O. Box 84428 Riyadh 11671 Saudi Arabia szalshawwa@pnu.edu.sa LMAlmutlaqah@pnu.edu.sa aaalrashidi@pnu.edu.sa; b Department of Pharmacology and Toxicology, Faculty of Medicine, Umm Al-Qura University Makkah Saudi Arabia gabamagous@uqu.edu.sa; c Department of General Surgery, Faculty of Medicine, Umm Al-Qura University Makkah 24382 Saudi Arabia hasembawa@uqu.edu.sa; d Department of Pharmaceutical Practice, College of Pharmacy, Princess Nourah Bint Abdulrahman University P. O. Box 84428 Riyadh 11671 Saudi Arabia almalkhodair@pnu.edu.sa; e Chemistry Department, Faculty of Science, Suez Canal University 41522 Ismailia Egypt essa.saied@science.suez.edu.eg; f Institute for Chemistry, Humboldt Universität zu Berlin 12489 Berlin Germany saiedess@hu-berlin.de

## Abstract

A set of quinoxaline-based derivatives incorporating a cyanoacrylate pharmacophore were designed, synthesized, and biologically evaluated for their dual anticancer and anti-inflammatory potential. Cytotoxic activity was assessed against HepG-2, HCT-116, MCF-7, and Panc-1 cancer cell lines using the MTT assay. Among the synthesized compounds, compound 9 exhibited the highest potency, particularly against HepG-2 cells (IC_50_ = 7.81 ± 1.1 µM), surpassing doxorubicin (IC_50_ = 15.96 ± 0.61 µM), and demonstrated improved selectivity toward WI-38 normal fibroblasts (IC_50_ = 67.21 µM; SI ≈ 7.81). Mechanistic investigations in HepG-2 cells revealed that compound 9 induced pronounced G0/G1 cell cycle arrest, increasing the cell population from 54.39% to 86.21% (∼1.6-fold), with a concomitant reduction in S-phase cells (∼3.1-fold decrease). Apoptosis analysis showed a significant increase in total apoptotic cells from 3.12% to 35.14% (∼11.3-fold), predominantly driven by late apoptosis (∼129-fold increase). These findings were supported by gene expression analysis, where compound 9 upregulated p53 (∼5.9-fold), BAX (∼3.7-fold), cytochrome c (∼3.9-fold), and caspase-7 (∼2.6-fold), while downregulating Bcl-2 (∼0.64-fold), indicating activation of the intrinsic mitochondrial apoptotic pathway. In LPS-stimulated RAW264.7 macrophages, compound 9 exhibited potent anti-inflammatory activity without significant cytotoxicity. The compound markedly reduced intracellular reactive oxygen species (ROS) levels in a dose-dependent manner (from 591.52 to 60.62 pg mL^−1^; ∼9.8-fold reduction) and significantly suppressed nitric oxide production. Furthermore, it downregulated key pro-inflammatory cytokines, including TNF-α (∼0.36-fold), IL-1β (∼0.47-fold), and IL-6 (∼0.51-fold), with effects comparable to celecoxib. Molecular docking studies indicated favorable binding of compound 9 within the active sites of IL-1β, TNF-α, and IL-6Rα, with the highest affinity toward IL-6Rα (−7.93 kcal mol^−1^). These interactions were further validated by 100 ns molecular dynamics simulations, which supported stable protein–ligand complexes, consistent RMSD profiles, preserved structural compactness, and persistent key interactions throughout the trajectories. Collectively, these findings identify compound 9 as a promising multi-target quinoxaline-based agent with dual antiproliferative and anti-inflammatory activities, mediated through induction of mitochondrial apoptosis and modulation of oxidative stress and cytokine signaling pathways.

## Abstract 2

A set of quinoxaline-based derivatives incorporating a cyanoacrylate pharmacophore were designed, synthesized, and biologically evaluated for their dual anticancer and anti-inflammatory potential.

## Graphical abstract

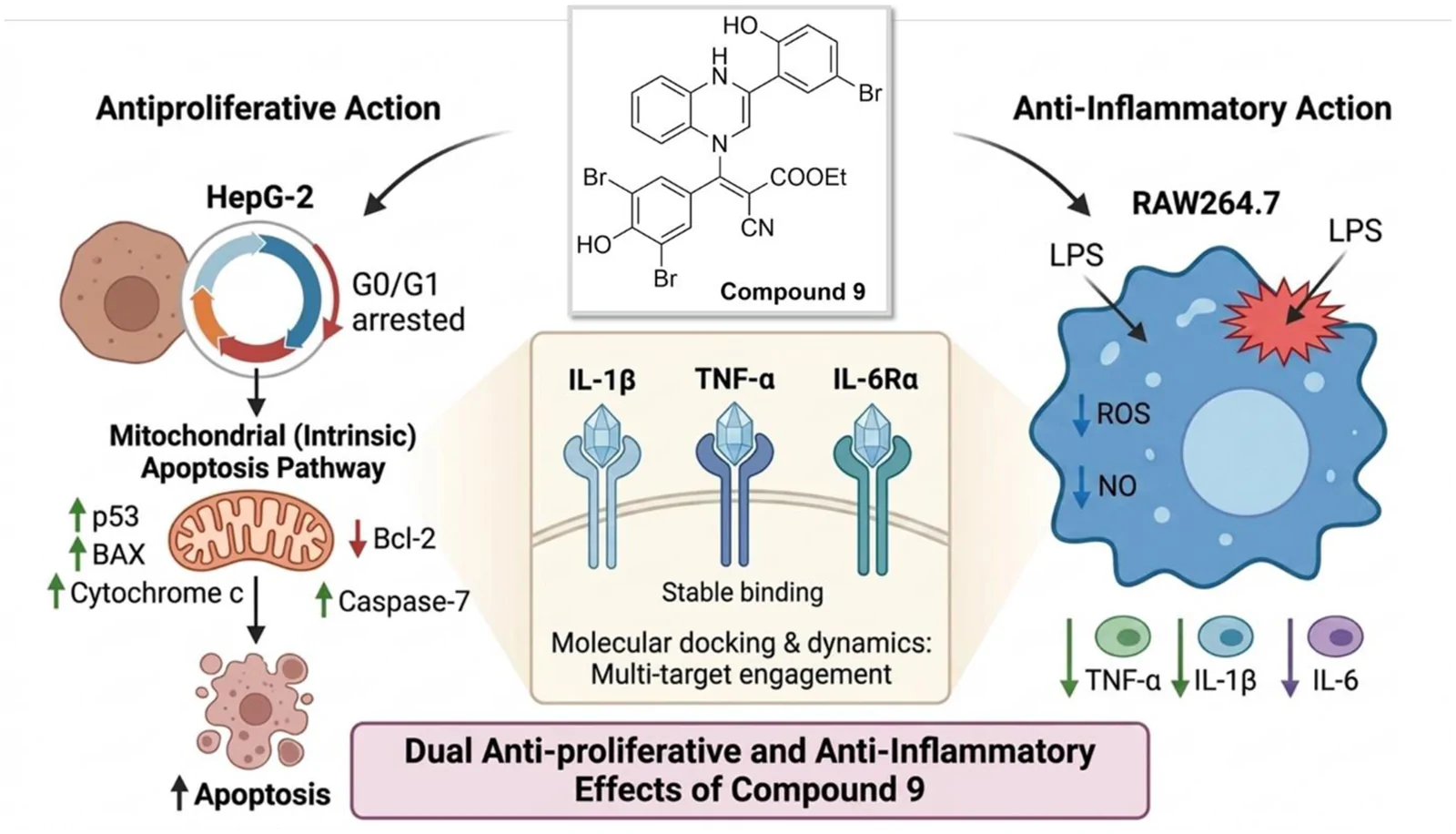

## 1. Introduction

Cancer remains one of the leading causes of mortality worldwide and represents a major challenge to global health. It is a complex and multifactorial disease characterized by uncontrolled cell proliferation and the ability of malignant cells to invade and metastasize to distant tissues.^1^ Both genetic predisposition and environmental factors contribute to cancer development, including viral infections such as human papillomavirus (HPV) and hepatitis B virus (HBV).^2^ Recent estimates indicate that approximately 1 958 310 new cancer cases and 609 820 cancer-related deaths were projected in the United States in 2023, emphasizing the urgent need for the development of more effective therapeutic strategies.^3^ Despite significant advances in cancer therapy, including chemotherapy, targeted therapy, and immunotherapy, current treatment strategies are often associated with severe side effects, drug resistance, and limited long-term efficacy.^4^ Current anticancer treatment relies on several major mechanistic classes, including DNA-damaging agents such as doxorubicin, antimitotic drugs targeting tubulin, kinase inhibitors directed against signaling proteins such as EGFR, VEGFR, CDKs, and PI3K, and immune-based approaches including PD-1/PD-L1 and CTLA-4 blockade. Although these strategies have improved outcomes in several malignancies, their clinical benefit is often limited by dose-limiting toxicity, tumor heterogeneity, intrinsic or acquired resistance, and incomplete suppression of the multiple pathways that sustain tumor growth.^5,6^ Accumulating evidence indicates that chronic inflammation plays a crucial role in cancer development and progression.^5^ Chronic inflammatory responses contribute to tumor initiation and progression through the production of reactive oxygen species (ROS), cytokines, and growth factors. In particular, pro-inflammatory cytokines such as tumor necrosis factor-α (TNF-α), interleukin-1β (IL-1β), and interleukin-6 (IL-6) play crucial roles in proliferation, angiogenesis, survival signaling, metastatic progression, and immune evasion, largely through pathways including nuclear factor kappa B (NF-κB).^7^ Accordingly, compounds capable of simultaneously modulating tumor cell survival and inflammatory signaling are increasingly viewed as attractive leads for next-generation anticancer drug discovery.

**Fig. 1:**
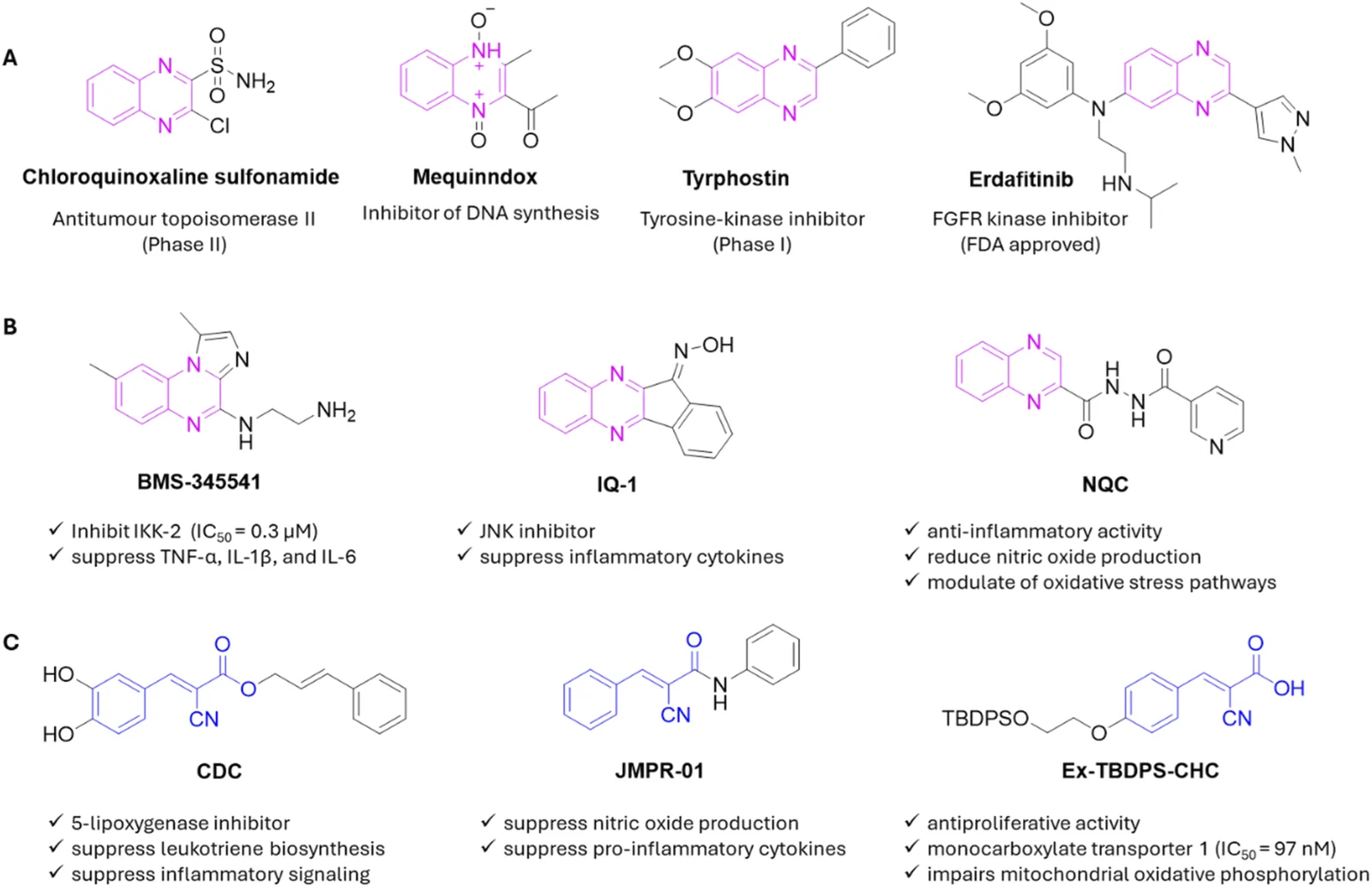
Structural basis for the design of quinoxaline–cyanocinnamate hybrids: representative clinically relevant quinoxaline scaffolds (A), anti-inflammatory quinoxaline derivatives (B), and α,β-unsaturated cyanoacrylate analogues with anti-inflammatory and anticancer activities (C).

In this context, quinoxaline derivatives have attracted considerable attention owing to their broad spectrum of biological activities, including anticancer, anti-inflammatory, antimicrobial, and kinase inhibitory properties.^8–10^ Several quinoxaline-containing compounds have reached clinical use or advanced preclinical evaluation, underscoring the pharmacological relevance of this scaffold in oncology (Fig. 1). Notably, erdafitinib, a quinoxaline-based molecule, has been approved as a fibroblast growth factor receptor (FGFR) inhibitor for the treatment of urothelial carcinoma. In addition to this clinically validated agent, a number of quinoxaline derivatives have demonstrated promising anticancer activity at the preclinical level. For instance, tyrphostin analogues, which incorporate a quinoxaline core, function as tyrosine kinase inhibitors targeting platelet-derived growth factor receptor (PDGFR) and have been investigated for brain tumor therapy. Furthermore, compounds such as mequinox (mequindox), reported as DNA synthesis inhibitors, and chloroquinoxaline sulfonamide derivatives, which act as topoisomerase IIα/IIβ inhibitors, have shown notable antiproliferative potential.^10^ The versatility of the quinoxaline scaffold in modulating multiple oncogenic pathways, including kinase signaling and DNA replication processes, thereby supporting its continued exploration in anticancer drug design. In this regard, numerous studies have demonstrated that quinoxaline-based compounds can induce apoptosis, modulate oxidative stress, and interfere with key signaling pathways involved in tumor progression.^11–14^ Quinoxaline derivatives have also demonstrated potent anti-inflammatory activity through suppressing nitric oxide production and inflammatory cytokines (TNF-α, IL-1β, and IL-6) in LPS-stimulated cells, thereby blocking NF-κB activation (*e.g.*, BMS-345541, IQ-1, NQC) (Fig. 1).^15–17^ Likewise, α,β-unsaturated cyanoacrylate derivatives have been widely explored as bioactive pharmacophores in drug design. These compounds are characterized by an electron-deficient olefinic system capable of interacting with biological nucleophiles, which may contribute to their cytotoxic and anti-inflammatory activities. Several reports have demonstrated that cyanoacrylate-containing molecules exhibit anti-inflammatory potential, primarily linked to their ability to modulate cellular metabolism (*e.g.*, Ex-TBDPS-CHC, and JMPR-01),^18,19^ and inflammatory signaling pathways (*e.g.*, CDC) (Fig. 1).^20^

From a design perspective, combining a quinoxaline core with an α,β-unsaturated cyanoacrylate pharmacophore is particularly appealing because it merges a scaffold known for target engagement in cancer and inflammation with an electrophilic fragment capable of enhancing target interactions and pathway modulation. Such a hybrid approach may provide broader pathway coverage than single-mechanism agents, especially in inflammation-driven malignancies where oxidative stress, cytokine signaling, angiogenesis, and apoptosis resistance are closely interconnected.^21–25^ Based on these considerations and our continuous efforts in discovering novel bioactive compounds,^26–30^ the present study describes the design and synthesis of a series of novel quinoxaline–cyanoacrylate hybrid derivatives. The synthesized compounds were evaluated for their cytotoxic activity against a panel of human cancer cell lines, followed by mechanistic investigations including cell cycle analysis, apoptosis induction, and gene expression profiling. In addition, their anti-inflammatory potential was assessed through evaluation of ROS generation, nitric oxide production, and cytokine expression in LPS-stimulated macrophages. To further elucidate the molecular basis of their activity, molecular docking and molecular dynamics simulation studies were performed against key inflammation-associated targets, including IL-1β, TNF-α, and IL-6 receptors.

## 2. Results and discussion

### 2.1. Chemistry

**Scheme 1:**
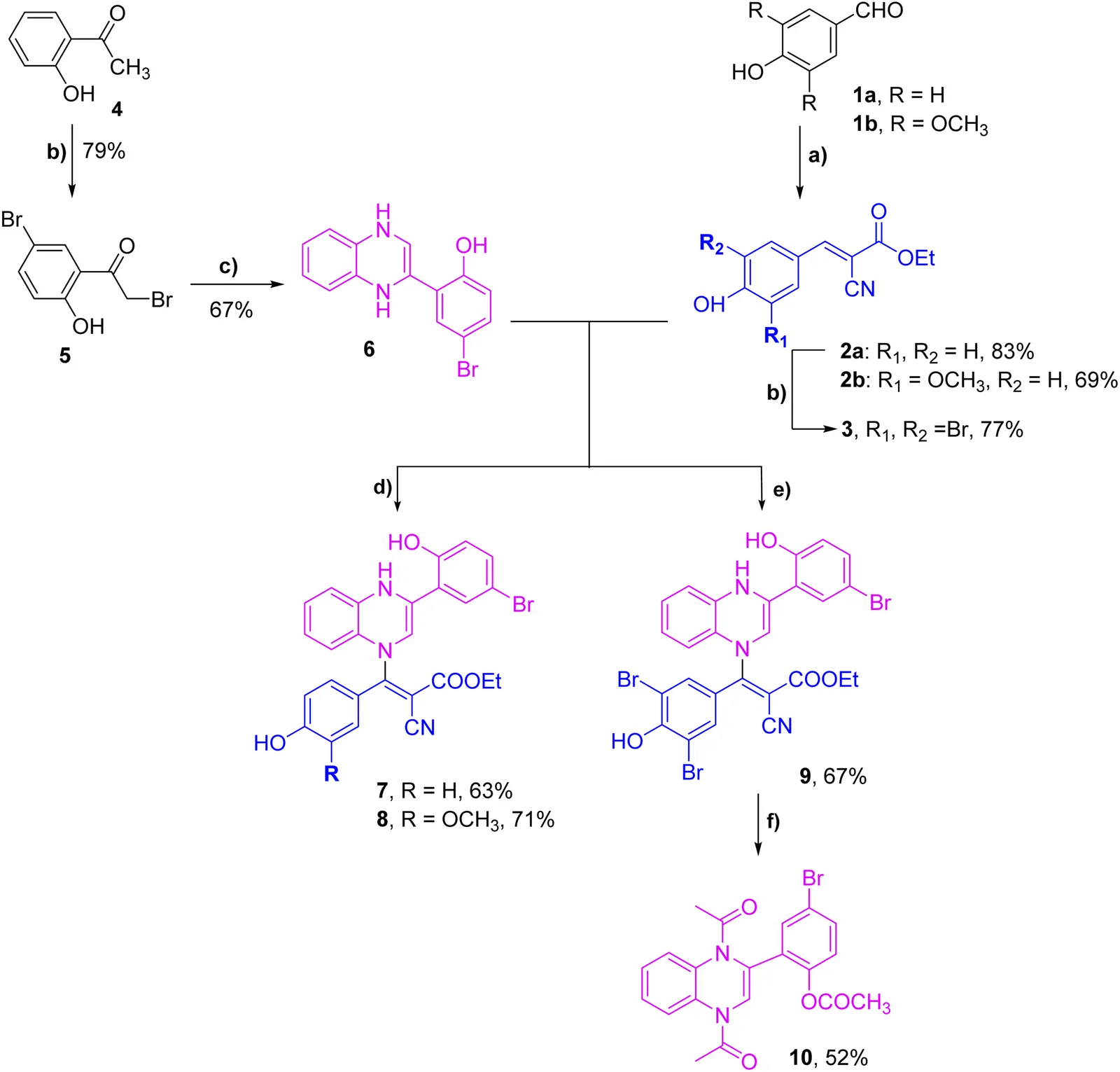
Synthesis of 1,3-disubstituted-1,4-dihydroquinoxaline–cyanoacrylate hybrids derivatives (6–10). Reagents and conditions: (a) cyanoacetic acid ethyl ester, pip. (cat), hexane, reflux at 68 °C, 16 h; (b) bromine, glacial acetic acid, 70 °C, 12–16 h; (c) *o*-PD, NaOAc, ethanol, 78 °C, 12 h; (d) i-2a/2b, K_2_CO_3_ anhyd, EtOH, 3 h; ii-6, 78 °C, 12–18 h; (e) i-3, K_2_CO_3_ anhyd, EtOH, 2 h; ii-6, 78 °C, 16 h; (f) acetic anhydride, 140 °C, 12 h.

The synthesis of the targeted 1,3-disubstituted-1,4-dihydroquinoxaline–cyanoacrylate hybrid derivatives was initiated from commercially available 4-hydroxybenzaldehydes (1a,b) and 2-hydroxyacetophenone (4), as outlined in Scheme 1. The ethyl 3-aryl-2-cyanoacrylate intermediates were prepared according to reported procedures *via* Knoevenagel condensation of 4-hydroxybenzaldehydes, namely 4-hydroxybenzaldehyde (1a) and 4-hydroxy-3-methoxybenzaldehyde (1b), with ethyl cyanoacetate in the presence of catalytic piperidine (pip.) at 68 °C, affording compounds 2a and 2b. Subsequent bromination of compound 2a was achieved by refluxing with bromine in glacial acetic acid, yielding ethyl 3-(3,5-dibromo-4-hydroxyphenyl)-2-cyanoacrylate (3) in 77% yield.^21^ In parallel, the 1,4-dihydroquinoxaline core was synthesized *via* a two-step sequence starting from 2-hydroxyacetophenone (4). Bromination of compound 4 afforded 5-bromo-2-hydroxyphenacyl bromide (5) in 79% yield. This intermediate underwent cyclocondensation with *o*-phenylenediamine (*o*-PD) in the presence of fused sodium acetate under reflux conditions to furnish 2-(5-bromo-2-hydroxyphenyl)-1,4-dihydroquinoxaline (6) in 67% yield (Scheme 1).^31^ With both key fragments in hand, the target hybrid molecules were constructed through nucleophilic substitution reactions. Treatment of compound 6 with the corresponding ethyl 3-aryl-2-cyanoacrylates (2a, 2b, and 3) in the presence of potassium carbonate under reflux conditions afforded the desired 1,3-disubstituted quinoxaline–cyanoacrylate hybrids (7–9) in moderate to good yields (63–71%). Interestingly, acetylation of compound 9 using acetic anhydride under reflux conditions resulted in an unexpected cleavage reaction rather than simple acetylation, leading to the formation of two distinct products: 1,4-diacetyl-2-(5-bromo-2-acetoxyphenyl)quinoxaline (10) and ethyl 3-(3,5-dibromo-4-acetoxyphenyl)-2-cyanoacrylate (11). This observation suggests a susceptibility of the hybrid linkage to acylation-induced fragmentation under the applied conditions. Since compound 11 was formed as an unexpected side product rather than a member of the intended quinoxaline–cyanoacrylate hybrid series, it was not included in the subsequent biological evaluation (chemical structure and spectroscopic characterization are provided in the SI). The structures of all synthesized compounds were confirmed by elemental analysis, FT-IR, ^1^H NMR, ^13^C NMR, and mass spectrometry. The ^1^H NMR spectra of compounds 7–9 exhibited characteristic singlet signals corresponding to the NH proton of the 1,4-dihydroquinoxaline moiety at *δ* 12.94, 12.95, and 11.73 ppm, respectively, confirming the retention of the reduced quinoxaline core. Additionally, phenolic hydroxyl protons were observed as singlet signals in the range *δ* 10.21–10.85 ppm, consistent with the presence of free OH groups in the respective structures. A key diagnostic feature was the appearance of a singlet signal corresponding to the methine proton of the quinoxaline ring (CH-quinoxaline) at *δ* 5.31, 5.40, and 5.60 ppm for compounds 7, 8, and 9, respectively, indicating successful substitution at the N-1 position and formation of the hybrid system. The aromatic region displayed multiplet signals in the expected range, consistent with the proposed substitution patterns. The ^13^C NMR spectra further corroborated the proposed structures, showing characteristic ester carbonyl signals at *δ* ∼162–163 ppm, along with a distinct signal for the cyano group at *δ* ∼110 ppm. Importantly, the methine carbon of the quinoxaline ring appeared consistently at *δ* ∼97.5 ppm in all compounds, serving as a key structural indicator of the dihydroquinoxaline framework. Additional supporting evidence was obtained from IR spectra, which displayed absorption bands corresponding to hydroxyl (≈3400 cm^−1^), NH (≈3220 cm^−1^), cyano (≈2220 cm^−1^), and ester carbonyl (≈1740–1750 cm^−1^) functionalities, confirming the presence of the cyanoacrylate moiety. Mass spectrometric analysis of compounds 7 and 8 provided further confirmation of their proposed structures. The spectra displayed molecular ion peaks at *m*/*z* 517 and 547, corresponding to the molecular formulas C_26_H_20_BrN_3_O_4_ and C_27_H_22_BrN_3_O_5_, respectively. These molecular ion peaks were relatively weak, indicating their inherent instability under electron ionization conditions (Fig. S1). Both compounds exhibited a common fragmentation pathway involving cleavage of the cyanoacrylate moiety, leading to the formation of a prominent fragment ion at *m*/*z* 301, which corresponds to the molecular ion radical cation of the quinoxaline core, namely 2-(5-bromo-2-hydroxyphenyl)-1,4-dihydroquinoxaline. This fragment underwent further loss of nitrogen to yield a stable ion at *m*/*z* 287. Subsequent fragmentation of the *m*/*z* 287 ion resulted in the formation of characteristic fragments at *m*/*z* 261 and 233, attributed to the successive elimination of cyano and carbonyl functionalities. An alternative fragmentation pathway involved cleavage of the quinoxaline moiety, generating fragment ions at *m*/*z* 216 and 247, corresponding to substituted cyanoacrylate fragments. Further fragmentation of these ions, through the loss of ethoxycarbonyl (CO_2_Et) and oxygen-containing groups, produced additional peaks at *m*/*z* 143, 173, 127, and 157. Notably, the base peak observed at *m*/*z* 216 in compound 7 is assigned to the ethyl β-(4-hydroxyphenyl)-α-cyanoacrylate cation, indicating its high stability under the applied ionization conditions.

The structures of the acetylated products 10 and 11 were confirmed by ^1^H and ^13^C NMR spectroscopy in addition to mass spectrometric analysis. For compound 10, the ^1^H NMR spectrum showed the complete disappearance of the characteristic NH and OH proton signals observed in the precursor, confirming successful acetylation. Instead, three new singlet signals appeared at *δ* 1.77, 1.80, and 2.11 ppm, corresponding to the methyl protons of three acetyl groups. This observation clearly indicates the formation of a triacetylated derivative. The ^13^C NMR spectrum further supported this assignment, displaying three distinct carbonyl carbon signals at *δ* 173.54, 172.06, and 167.87 ppm, attributable to the acetyl functionalities. In addition, three methyl carbon signals were observed at *δ* 24.06, 23.76, and 22.97 ppm, consistent with the presence of three acetyl groups. Mass spectrometric analysis of compound 10 revealed a fragmentation pattern consistent with its proposed structure. Notably, a fragment ion peak at *m*/*z* 302, corresponding to the molecular ion of compound 6, was observed, indicating cleavage of the acetylated structure to regenerate the quinoxaline core. This fragment underwent further decomposition through the loss of nitrogen and small neutral molecules (*e.g.*, CN and CO), affording characteristic fragment ions at *m*/*z* 289/287, 263/261, and 225/223, which further support the assigned structure. For compound 11, the ^1^H NMR spectrum displayed signals characteristic of the ethyl ester functionality, including a triplet at *δ* 1.27–1.31 ppm (3H, CH_3_) and a quartet at *δ* 4.26–4.32 ppm (2H, OCH_2_). Additionally, a singlet at *δ* 1.91 ppm was assigned to the methyl protons of the acetoxy group, confirming successful acetylation. The olefinic proton appeared as a singlet at *δ* 8.24 ppm, while the aromatic protons were observed as singlets around *δ* 8.29 ppm, consistent with the substituted aromatic system. The ^13^C NMR spectrum of compound 11 showed two carbonyl carbon signals at *δ* 172.62 and 162.25 ppm, corresponding to the ester and acetoxy groups, respectively. Signals at *δ* 62.84, 21.50, and 14.43 ppm were assigned to the ethoxy (OCH_2_CH_3_) and acetyl methyl carbons. Furthermore, the cyano and C–O carbons appeared at *δ* 101.40 and 155.82 ppm, respectively, supporting the presence of the cyanoacrylate framework.

### 2.2. Assessment of cytotoxic potential

**Fig. 2:**
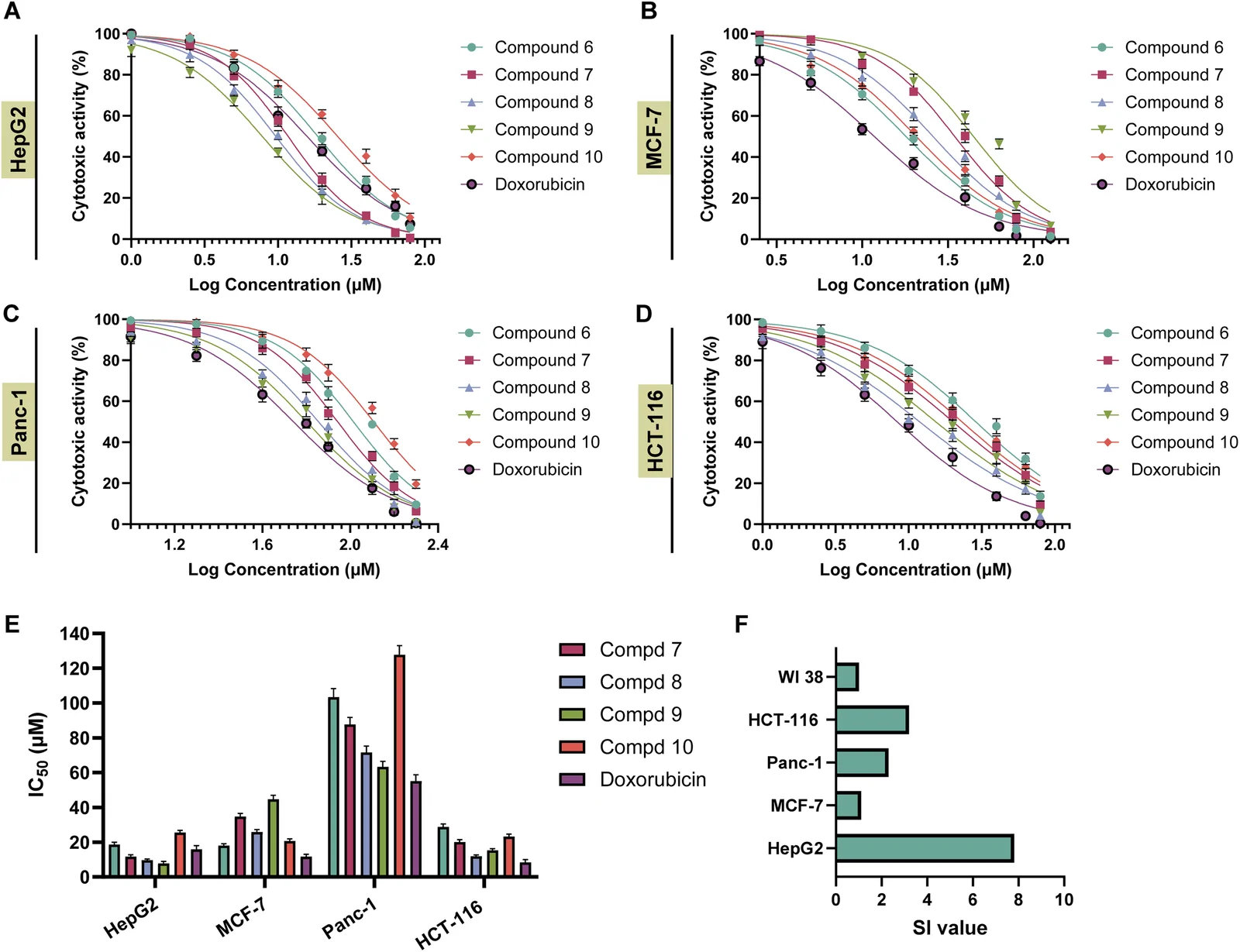
Cytotoxic activity of the synthesized quinoxaline derivatives (6–10) evaluated against different human cancer cell lines using the MTT assay. Dose–response curves representing the effect of increasing concentrations (log µM) on cell viability (%) are shown for (A) HepG2, (B) MCF-7, (C) Panc-1, and (D) HCT-116 cells. (E) Comparative IC_50_ values (µM) of the tested compounds and the reference drug doxorubicin across the examined cell lines. (F) Selectivity index (SI) values of compound 9 relative to WI-38 normal human fibroblasts. Data are expressed as mean ± SD, *n* = 3.

The cytotoxic activity of the synthesized quinoxaline derivatives (6–10) was evaluated against a panel of human cancer cell lines, including HepG-2 (hepatoblastoma), HCT-116 (colon), MCF-7 (breast), and Panc-1 (pancreatic), using the MTT assay, and compared with the reference drug doxorubicin. The results revealed a clear structure–activity relationship, influenced by the incorporation of the 2-cyano-3-aryl acrylate moiety and the nature of substituents on the aryl ring, with distinct cell line–dependent responses (Fig. 2). In HepG-2 cells, the parent compound 6 exhibited moderate activity (IC_50_ = 18.65 ± 1.4 µM), slightly weaker than doxorubicin (15.96 ± 0.61 µM). Introduction of the cyanoacrylate pharmacophore significantly enhanced activity, as observed for compounds 7–9, with IC_50_ values of 11.71 ± 1.1, 9.66 ± 0.7, and 7.81 ± 1.1 µM, respectively. Compound 9 emerged as the most potent derivative, surpassing doxorubicin, which can be attributed to the presence of 3,5-dibromo substituents that enhance lipophilicity and potential halogen bonding interactions. In contrast, acetylated compound 10 showed reduced activity (25.49 ± 1.6 µM), highlighting the importance of free phenolic groups for optimal biological interaction. It is important to note that all cytotoxicity experiments in the present study were conducted following 24 h exposure. The cytotoxic activity of doxorubicin is known to be highly time-dependent, and substantially lower IC_50_ values are frequently reported after longer incubation periods (48–72 h) due to increased intracellular accumulation and enhanced induction of cell death pathways. Notably, several previous studies have reported doxorubicin IC_50_ values within a comparable micromolar range following 24 h treatment, supporting the validity of the present findings.^32–35^ A different activity trend was observed in MCF-7 cells, where compound 6 showed moderate activity (IC_50_ = 18.02 ± 1.1 µM), while derivatives 7–9 displayed reduced potency (IC_50_ = 34.67 ± 1.9, 25.89 ± 1.4, and 44.66 ± 2.3 µM, respectively), indicating that the introduction of the cyanoacrylate moiety is not favorable in this cell line. Interestingly, compound 10 (IC_50_ = 20.69 ± 1.2 µM) restored activity comparable to the parent scaffold, suggesting that increased lipophilicity *via* acetylation may partially compensate for the loss of hydrogen bonding in this context. Nevertheless, all compounds were less potent than doxorubicin (IC_50_ = 11.76 ± 0.61 µM), indicating that MCF-7 cells have limited sensitivity to this scaffold modification. In Panc-1 cells, all compounds exhibited relatively weak cytotoxic activity, with IC_50_ values ranging from 63.24 ± 3.2 to 127.8 ± 5.3 µM, compared to doxorubicin (55.09 ± 0.61 µM). Compound 9 remained the most active (63.24 ± 3.2 µM), maintaining the trend observed in HepG-2 cells, which suggests that halogen substitution enhances activity even in relatively resistant cell lines. In HCT-116 cells, a notable improvement in activity was observed upon structural modification. The parent compound 6 showed moderate activity (IC_50_ = 28.78 ± 1.72 µM), while compound 7 improved to 20.11 ± 1.3 µM. A significant enhancement was achieved with compound 8 (IC_50_ = 11.89 ± 0.8 µM), indicating that the presence of both hydroxyl and methoxy substituents plays a favorable role, likely through electronic and steric effects that enhance interaction with cellular targets. Compound 9 (IC_50_ = 15.34 ± 0.9 µM) also showed strong activity, though slightly less potent than compound 8, suggesting that excessive halogenation may introduce steric constraints. Compound 10 showed reduced activity (23.15 ± 1.5 µM), again confirming the importance of free phenolic groups. Despite these improvements, doxorubicin remained more potent (IC_50_ = 8.35 ± 0.61 µM) (Fig. 2).

These results reveal a consistent SAR across the tested cancer cell lines dictated by a delicate interplay between electrophilicity, hydrogen bonding capacity, lipophilicity, and steric factors, with the cyanoacrylate moiety and strategic aryl substitution being critical determinants of potency and cell line selectivity. The parent quinoxaline scaffold (compound 6) exhibited moderate activity across most cell lines, indicating that while the quinoxaline core contributes to baseline cytotoxicity, it requires further functionalization for enhanced potency. Introduction of the cyanoacrylate fragment in compounds 7–9 generally improved activity, particularly in HepG-2 and HCT-116 cells, highlighting the importance of the α,β-unsaturated system as a key pharmacophoric element. Substituent effects on the aryl ring also played a role in modulating activity and selectivity across cell lines. The presence of a free phenolic hydroxyl group (compound 7) contributed to improved activity, likely through hydrogen bonding interactions, while further substitution with a methoxy group (compound 8) enhanced activity, particularly in HCT-116 cells, suggesting that electron-donating groups increase electron density and stabilize the conjugated system, thereby strengthening ligand–target interactions. In contrast, incorporation of electron-withdrawing bromine atoms (compound 9) significantly enhanced activity in HepG-2 and Panc-1 cells, indicating that increased lipophilicity and electrophilicity favor activity in these cell types, possibly through improved membrane permeability and improved interactions such as halogen bonding. Interestingly, the SAR trend observed in MCF-7 cells deviated from that of other cell lines, where the introduction of the cyanoacrylate moiety (compounds 7–9) led to reduced activity compared to the parent scaffold. This suggests that increased electrophilicity or molecular size may not be favorable in this cell line, potentially due to differences in cellular uptake mechanisms. In this context, the acetylated derivative (compound 10) showed comparable activity to compound 6, indicating that increased lipophilicity may partially compensate for the loss of hydrogen bonding capability. However, across HepG-2 and HCT-116 cells, acetylation resulted in reduced activity, confirming that free phenolic groups are essential for optimal interaction with biological targets. Although the present series provides valuable preliminary insights into the influence of electron-donating, electron-withdrawing, and hydrogen-bonding substituents on the quinoxaline–cyanoacrylate scaffold, a broader analogue library incorporating additional substitution patterns would enable a more comprehensive understanding of the electronic and steric requirements governing biological activity.

Based on these results, compound 9, among the tested derivatives, exhibited the highest potency across different cancer cell lines. Although its activity was not uniformly superior in all evaluated models, it consistently ranked among the most active compounds in most of the tested cancer cell lines. Accordingly, compound 9 was further evaluated against WI-38 normal human fibroblasts to assess its selectivity profile in comparison with doxorubicin (Fig. S2). Compound 9 exhibited an IC_50_ value of 67.2 µM toward WI-38 cells, compared with 59.6 µM for doxorubicin, indicating relatively lower cytotoxicity toward normal cells. The calculated selectivity index (SI) values further supported the favorable profile of compound 9. It demonstrated the highest selectivity toward HepG2 cells (SI = 7.81), followed by HCT-116 cells (SI = 3.20) and Panc-1 cells (SI = 2.30), while lower selectivity was observed against MCF-7 cells (SI = 1.10). In contrast, doxorubicin showed comparatively lower selectivity toward HepG2 cells (SI = 2.11), moderate selectivity toward MCF-7 (SI = 1.21) and HCT-116 cells (SI = 1.61), and higher selectivity toward Panc-1 cells (SI = 3.61) (Fig. 2F). These results are in agreement with previous reports, which showed that quinoxaline-based compounds exhibit potential antiproliferative activity across different cancer cells.^36–39^ Our findings indicate that compound 9 possesses a particularly favorable therapeutic window against HepG2 cells, where it combined the strongest cytotoxic potency with the highest degree of selectivity over normal fibroblasts. Notably, the selectivity of compound 9 toward HepG2 cells was substantially higher than that of doxorubicin, whereas its selectivity toward HCT-116 cells, although still acceptable, was lower than that of the reference drug. On the other hand, the low SI values observed in MCF-7 and Panc-1 cells suggest limited tumor selectivity in these models. Accordingly, HepG2 cells were selected as the principal model for subsequent mechanistic studies, since compound 9 displayed in this cell line is not only the strongest antiproliferative activity but also the most favorable selectivity profile among the tested cancer models.

### 2.3. Assessment of cell cycle arrest

**Fig. 3:**
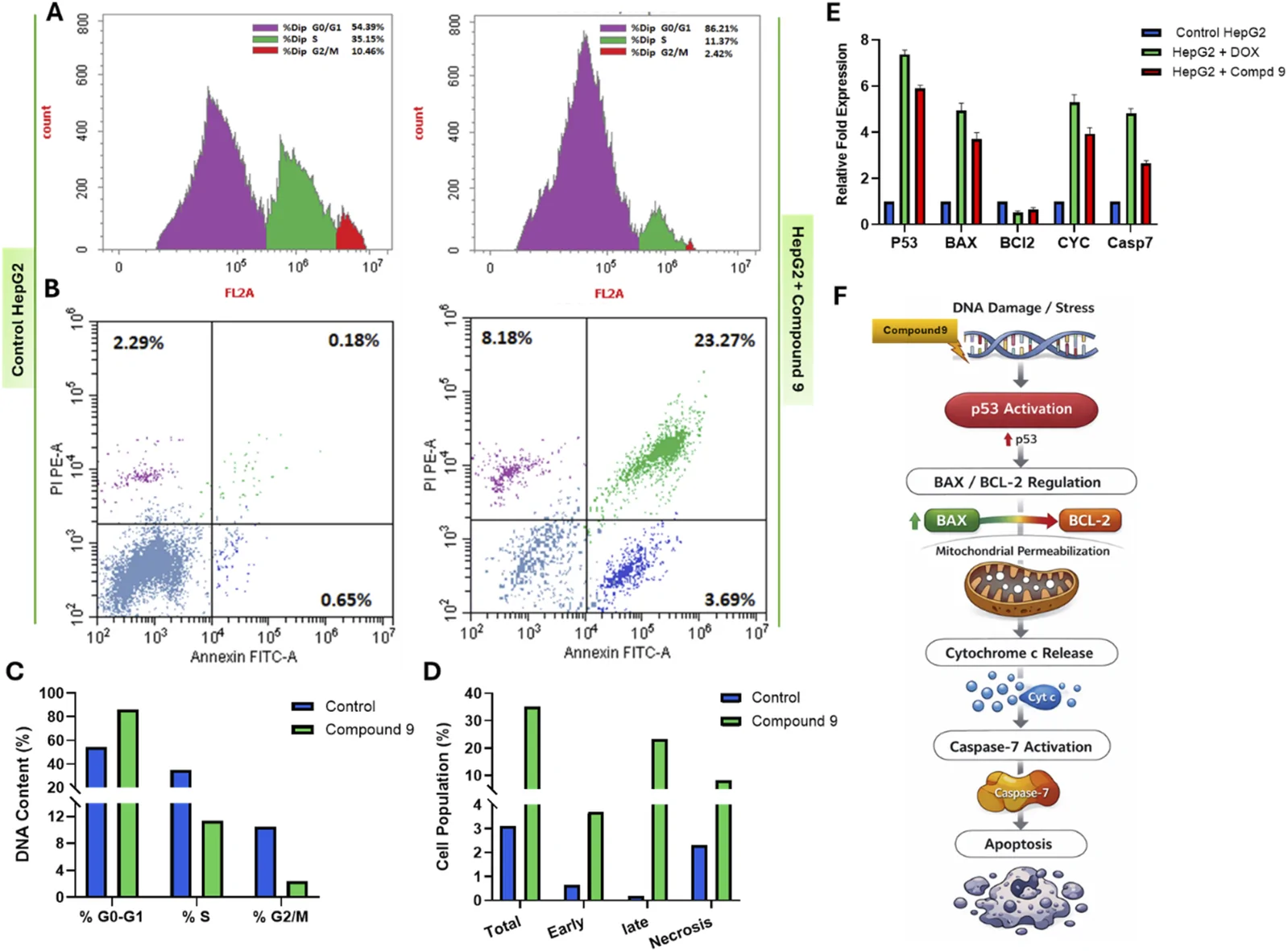
Mechanistic evaluation of compound 9 in HepG2 cells. (A) Cell cycle distribution analysis determined by flow cytometry showing the effect of compound 9 (at IC_50_) on G0/G1, S, and G2/M phases compared to control cells, indicating G0/G1 phase arrest. (B) Annexin V-FITC/PI staining assay illustrating apoptosis induction in HepG2 cells following treatment with compound 9, with increased early and late apoptotic populations compared to control. (C) Quantitative analysis of cell-cycle distribution, showing the percentages of cells in the G0/G1, S, and G2/M phases following treatment with compound 9 compared with untreated control cells. (D) Quantitative analysis of Annexin V-FITC/PI staining, showing the percentages of viable, early apoptotic, late apoptotic, and necrotic cells following treatment with compound 9 compared with untreated control cells. (E) Relative mRNA expression levels of apoptosis-related genes (p53, BAX, Bcl-2, cytochrome c, and caspase-7) in HepG2 cells treated with compound 9 compared to doxorubicin and control cells (data are expressed as mean ± SD, *n* = 3). (F) Proposed mechanism of action of compound 9, involving activation of the p53 pathway, modulation of BAX/Bcl-2 balance, mitochondrial membrane permeabilization, cytochrome c release, caspase-7 activation, and induction of apoptosis.

To explore the antiproliferative mechanism of compound 9, cell cycle distribution analysis was performed in HepG2 cells following treatment at its IC_50_ concentration, and DNA content was quantified using flow cytometry. As shown in Fig. 3A, treatment with compound 9 induced a marked alteration in cell cycle progression compared to untreated control cells. Specifically, compound 9 caused a pronounced accumulation of cells in the G0/G1 phase, increasing from 54.39% in control cells to 86.21%, corresponding to approximately a 1.58-fold increase. In parallel, a significant reduction in the S phase population was observed, decreasing from 35.15% to 11.37% (∼3.1-fold decrease), indicating strong inhibition of DNA synthesis. Similarly, the G2/M phase fraction declined from 10.46% to 2.42% (∼4.3-fold decrease), further supporting the suppression of cell cycle progression beyond the G1 checkpoint. This shift in cell cycle distribution clearly indicates that compound 9 induces cell cycle arrest at the G0/G1 phase, thereby preventing entry into the S phase and subsequent DNA replication. Such an effect is commonly associated with the modulation of key regulatory proteins controlling the G1 checkpoint, including tumor suppressor pathways such as p53. The observed depletion of S and G2/M populations further indicates that compound 9 effectively halts cell cycle progression at an early stage, ultimately leading to growth inhibition (Fig. 3A and C). Several studies have shown that quinoxaline-based compounds exhibit the ability to arrest cancer cells in different phases.^40–42^ Overall, these findings reveal that the antiproliferative activity of compound 9 in HepG2 cells is mediated, at least in part, through induction of G0/G1 phase arrest, providing mechanistic insight into its cytotoxic effect and supporting its potential as a cell cycle-targeting anticancer agent. It should be noted that the cell cycle analysis was conducted at the IC_50_ concentration of compound 9, which was selected as a biologically relevant concentration for mechanistic evaluation. While assessment of additional concentrations may provide further information regarding the dose-dependency of cell cycle modulation, the present data clearly demonstrate the ability of compound 9 to induce significant G0/G1 phase arrest under cytotoxic conditions.

### 2.4. Assessment of apoptosis induction potential

To investigate whether the cytotoxic effect of compound 9 is associated with apoptosis induction, Annexin V-FITC/PI staining was performed in HepG2 cells following treatment at its IC_50_ concentration, and apoptotic cell populations were quantified by flow cytometry. The results revealed a pronounced increase in apoptotic cell death upon treatment with compound 9 compared to untreated control cells (Fig. 3B and D). The total apoptotic population (early + late apoptosis) increased from 3.12% in control cells to 35.14% following treatment, corresponding to approximately an 11.3-fold increase, indicating strong induction of apoptosis. This effect was primarily driven by a substantial elevation in late apoptosis, which increased from 0.18% to 23.27% (∼129-fold increase), suggesting progression of cells toward irreversible apoptotic stages. In contrast, early apoptosis showed a moderate increase from 0.65% to 3.69% (∼5.7-fold increase), indicating that a significant proportion of cells had already transitioned beyond the early apoptotic phase at the time of analysis. Additionally, necrotic cell death increased from 2.29% to 8.18% (∼3.6-fold increase), although this remained markedly lower than the apoptotic fraction, suggesting that apoptosis is the predominant mode of cell death induced by compound 9. This observation is consistent with the previously observed G0/G1 cell cycle arrest, indicating that disruption of cell cycle progression is followed by activation of programmed cell death pathways. The relatively lower proportion of early apoptotic cells further supports the notion of efficient and rapid execution of apoptosis rather than prolonged arrest in the early phase.

Next, to further explore the molecular mechanism underlying apoptosis induction by compound 9, the expression levels of key apoptotic markers were assessed in HepG2 cells following treatment at its IC_50_ concentration and compared with the reference drug doxorubicin (Fig. 3E). The results revealed significant modulation of both pro-apoptotic and anti-apoptotic genes, indicating activation of the intrinsic (mitochondrial) apoptotic pathway. Treatment with compound 9 resulted in a marked upregulation of p53, with expression increasing to 5.89-fold relative to control (*vs.* 7.38-fold for doxorubicin), indicating activation of the p53-mediated apoptotic signaling pathway. Similarly, the pro-apoptotic gene BAX was significantly upregulated to 3.71-fold (*vs.* 4.95-fold for doxorubicin), supporting the induction of mitochondrial apoptosis. In contrast, the anti-apoptotic gene Bcl-2 was downregulated to 0.64-fold relative to control (*vs.* 0.52-fold for doxorubicin), reflecting suppression of survival signaling. Notably, the BAX/Bcl-2 ratio, a critical determinant of apoptotic commitment, was markedly increased following treatment with compound 9 (∼5.8-fold increase over control), indicating a significant shift toward a pro-apoptotic cellular state (compared to ∼9.5-fold for doxorubicin). Furthermore, compound 9 significantly elevated the expression of cytochrome c (CYC) to 3.94-fold (*vs.* 5.3-fold for doxorubicin), indicating mitochondrial membrane permeabilization and release of apoptogenic factors. This was accompanied by upregulation of caspase-7 to 2.65-fold (*vs.* 4.8-fold for doxorubicin), supporting activation of downstream executioner caspases. These findings are in agreement with previous studies, which revealed that quinoxaline-based compounds display antiproliferative activity by triggering mitochondrial apoptosis.^40,41,43,44^ Together, these findings provide a coherent mechanistic framework for the antiproliferative activity of compound 9 in HepG2 cells. The pronounced accumulation of cells in the G0/G1 phase, accompanied by a marked depletion of S and G2/M populations, indicates that the cell cycle blockade is closely associated with activation of apoptotic pathways, as evidenced by the substantial increase in total apoptotic cells. At the molecular level, compound 9 triggered apoptosis through activation of the p53-dependent pathway, as shown by significant upregulation of p53 and its downstream pro-apoptotic effector BAX, coupled with suppression of the anti-apoptotic protein Bcl-2, resulting in a pronounced increase in the BAX/Bcl-2 ratio. This shift in the balance of apoptotic regulators promoted mitochondrial outer membrane permeabilization, leading to the release of cytochrome c and subsequent activation of executioner caspase-7. The convergence of these events clearly indicates that compound 9 exerts its cytotoxic effect predominantly through the induction of intrinsic apoptosis (Fig. 3F).

### 2.5. Assessment of anti-inflammatory potential

**Fig. 4:**
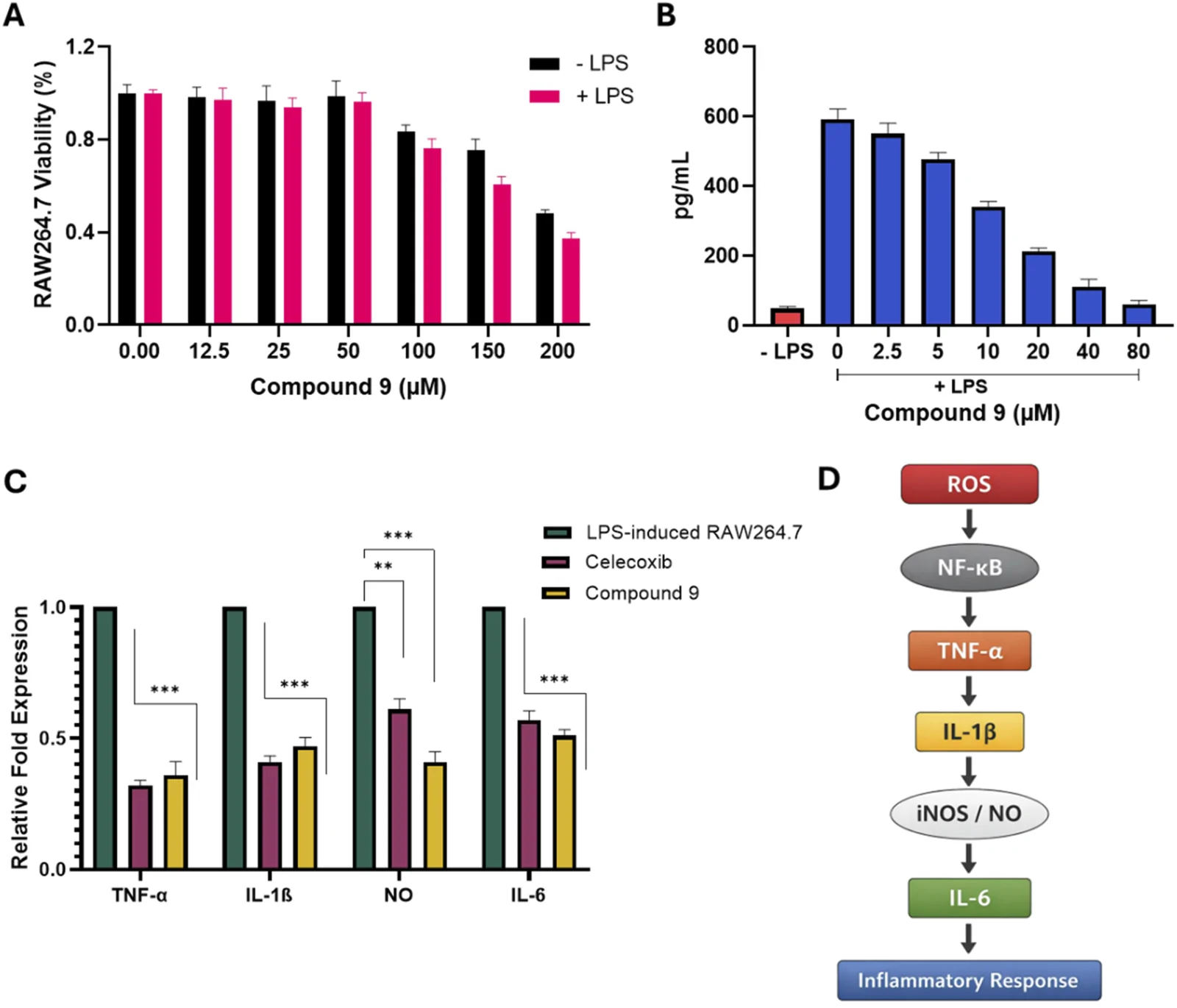
Evaluation of the anti-inflammatory activity of compound 9 in LPS-induced RAW 264.7 macrophages. (A) Cell viability assessment using the MTT assay showing the effect of compound 9 at different concentrations (µM) in the absence and presence of LPS stimulation (data are expressed as mean ± SD, *n* = 3). (B) Effect of compound 9 on intracellular reactive oxygen species (ROS) production, demonstrating a concentration-dependent reduction of ROS levels in LPS-stimulated cells (data are expressed as mean ± SD, *n* = 3). (C) Inhibitory effect of compound 9 on pro-inflammatory mediators (TNF-α, IL-1β, IL-6, and NO) compared with the reference drug celecoxib (data are expressed as mean ± SD, *n* = 3). (D) Proposed anti-inflammatory mechanism of compound 9, illustrating suppression of ROS generation and subsequent reduced expression of inflammatory cytokines and mediators.

Given the well-established link between inflammation and cancer progression, particularly in hepatic malignancies,^45,46^ the anti-inflammatory potential of compound 9 was further investigated. Initially, its cytotoxicity was evaluated in murine macrophage RAW 264.7 cells using the MTT assay in the absence and presence of lipopolysaccharide (LPS)-induced inflammatory conditions (Fig. 4A). The results indicated that compound 9 exhibited low to moderate cytotoxicity toward RAW 264.7 cells across the tested concentration range (12.5–200 µM) under both basal and inflammatory conditions. In the absence of LPS, compound 9 maintained high cell viability at lower concentrations, with viability values of 98.3%, 96.7%, and 98.6% at 12.5, 25, and 50 µM, respectively, indicating minimal cytotoxicity in non-stimulated macrophages. A gradual decline in cell viability was observed at higher concentrations, reaching 83.6%, 75.4%, and 48.1% at 100, 150, and 200 µM, respectively, suggesting dose-dependent cytotoxic effects at elevated concentrations. A similar trend was observed under LPS-stimulated conditions, where cell viability remained relatively high at lower concentrations (97.1%, 93.8%, and 96.4% at 12.5, 25, and 50 µM, respectively), followed by a moderate decrease at higher concentrations (76.3%, 60.7%, and 37.2% at 100, 150, and 200 µM, respectively). The comparable viability profiles observed in the presence and absence of LPS in RAW 264.7 cells indicate that compound 9 does not exhibit enhanced cytotoxicity under inflammatory conditions, ensuring effective evaluation of anti-inflammatory activity under LPS-induced conditions. Initially, the anti-inflammatory activity of compound 9 was evaluated by assessing its effect on intracellular reactive oxygen species (ROS) production in LPS-induced RAW 264.7 macrophages across a range of concentrations (2.5–80 µM). As shown in Fig. 4B, LPS stimulation markedly elevated ROS levels from a basal value of 48.23 pg mL^−1^ to 591.52 pg mL^−1^, supporting successful induction of oxidative stress in activated macrophages.^47^ Treatment with compound 9 resulted in a concentration-dependent reduction in ROS production, indicating a pronounced antioxidant effect. At lower concentrations, ROS levels were slightly reduced to 551.28 pg mL^−1^ (2.5 µM) and 477.75 pg mL^−1^ (5 µM), corresponding to approximately 7% and 19% reduction, respectively, relative to LPS-treated cells. A more substantial decrease was observed at intermediate concentrations, where ROS levels declined to 341.72 pg mL^−1^ (10 µM) and 212.85 pg mL^−1^ (20 µM), reflecting approximately 42% and 64% reduction, respectively. At higher concentrations, compound 9 exhibited strong ROS-suppressive activity, reducing ROS levels to 111.73 pg mL^−1^ (40 µM) and 60.62 pg mL^−1^ (80 µM), corresponding to approximately 81% and 90% reduction, approaching near-complete suppression of LPS-induced oxidative stress. These findings indicate that compound 9 effectively attenuates oxidative stress in activated macrophages. Given that ROS act as key upstream signaling molecules that trigger inflammatory cascades, particularly through activation of transcription factors such as NF-κB,^48^ this suppression suggests that compound 9 may interfere with early events in the inflammatory signaling pathway. Moreover, the maintained cell viability at these concentrations indicated that the observed antioxidant effect is not due to nonspecific cytotoxicity but reflects a genuine modulation of cellular oxidative status.

To explore the ROS-induced inflammatory signaling pathway, the effect of compound 9 (at 10 µM) on the expression of key pro-inflammatory mediators was evaluated in LPS-induced RAW 264.7 macrophages and compared with the reference drug celecoxib (at 10 µM). As shown in Fig. 4C, the results indicated that compound 9 effectively suppressed the expression of major inflammatory cytokines and mediators, including TNF-α, IL-1β, IL-6, and NO, confirming its broad anti-inflammatory activity. Specifically, treatment with compound 9 reduced TNF-α expression to 0.36-fold relative to LPS-stimulated control cells (*vs.* 0.32-fold for celecoxib), indicating strong inhibition of this key pro-inflammatory cytokine. Similarly, IL-1β expression was decreased to 0.47-fold (*vs.* 0.41-fold for celecoxib), while IL-6 levels were reduced to 0.51-fold (*vs.* 0.57-fold for celecoxib). In addition, compound 9 significantly suppressed NO levels to 0.409-fold, which is more pronounced than that observed with celecoxib (0.61-fold), highlighting its strong inhibitory effect on nitric oxide-mediated inflammatory signaling. These findings are consistent with the observed attenuation of ROS levels, suggesting that the anti-inflammatory activity of compound 9 is mediated through modulation of oxidative stress-dependent signaling pathways. Since ROS act as upstream regulators of inflammatory responses, their suppression likely leads to inhibition of downstream inflammatory mediators, including TNF-α, IL-1β, IL-6, and iNOS-derived NO. These findings are in agreement with previous studies, which indicated that quinoxaline analogues exhibit potential anti-inflammatory activity by modulating oxidative stress pathways (Fig. 4D).^49–53^ Collectively, the anti-inflammatory data indicate that compound 9 exerts its effects through a multi-level mechanism, involving suppression of ROS generation, followed by inhibition of pro-inflammatory cytokine production and nitric oxide release. This integrated mode of action, together with its previously revealed antiproliferative effects in HepG2 cells, supports the potential of compound 9 as a dual-function agent capable of targeting both oxidative stress and inflammation-driven cancer progression.

### 2.6. Computational analysis

#### 2.6.1 Molecular docking study

The reliability of the docking protocol was first validated by re-docking the co-crystallized ligands into their respective binding sites. For IL-1β (PDB ID: 8C3U), the native ligand was successfully reproduced within the active site, yielding a binding free energy of −9.46 kcal mol^−1^ and a root-mean-square deviation (RMSD) of 0.26 Å relative to the crystallographic conformation (Table 1). This low RMSD value confirms excellent agreement between the predicted and experimental binding modes, thereby validating the docking setup. Similarly, re-docking of the co-crystallized inhibitor into TNF-α (PDB ID: 2AZ5) resulted in a binding free energy of −6.13 kcal mol^−1^ and an RMSD of 0.79 Å, further supporting the reliability of the applied protocol. The reproduced binding mode preserved key interactions, particularly aromatic and electrostatic contacts with Tyr119, a residue critical for ligand stabilization within the TNF-α binding pocket (Table 1 and Fig. S3). In the case of IL-6Rα (PDB ID: 1N26), which lacks a co-crystallized small-molecule ligand, docking validation was performed using a previously reported reference antagonist. The reference compound exhibited a binding free energy of −6.19 kcal mol^−1^ and formed interactions consistent with literature-reported binding behavior, particularly within the IL-6 recognition interface. These interactions included hydrogen bonding and hydrophobic contacts with residues such as Leu123 and Leu90 (Table 1 and Fig. S3). Collectively, these validation results confirm that the adopted docking protocol is capable of accurately reproducing ligand binding across structurally diverse inflammatory targets.^54–56^

**Table 1:** Summary of molecular docking scores and key interacting residues for the co-crystallized ligands/reference antagonist and compound 9 across IL-1β, TNF-α, and IL-6Rα

Target	Co-crystallized ligand		Comp. 9
IL-1β	Docking score (kcal mol^−1^)	−9.46	−6.31
		RMSD = 0.26	
	Residues	Glu50, Lys93, Met95, Asn102, Lys94	Lys94, Lys97
TNF-α	Docking score (kcal mol^−1^)	−6.13	−5.73
		RMSD = 0.79	
	Residues	Tyr119	Tyr119
IL-6Rα	Docking score (kcal mol^−1^)	−6.19	−7.93
	Residues	Leu123, Leu90	Leu123, Leu90, Leu69, Leu89

**Fig. 5:**
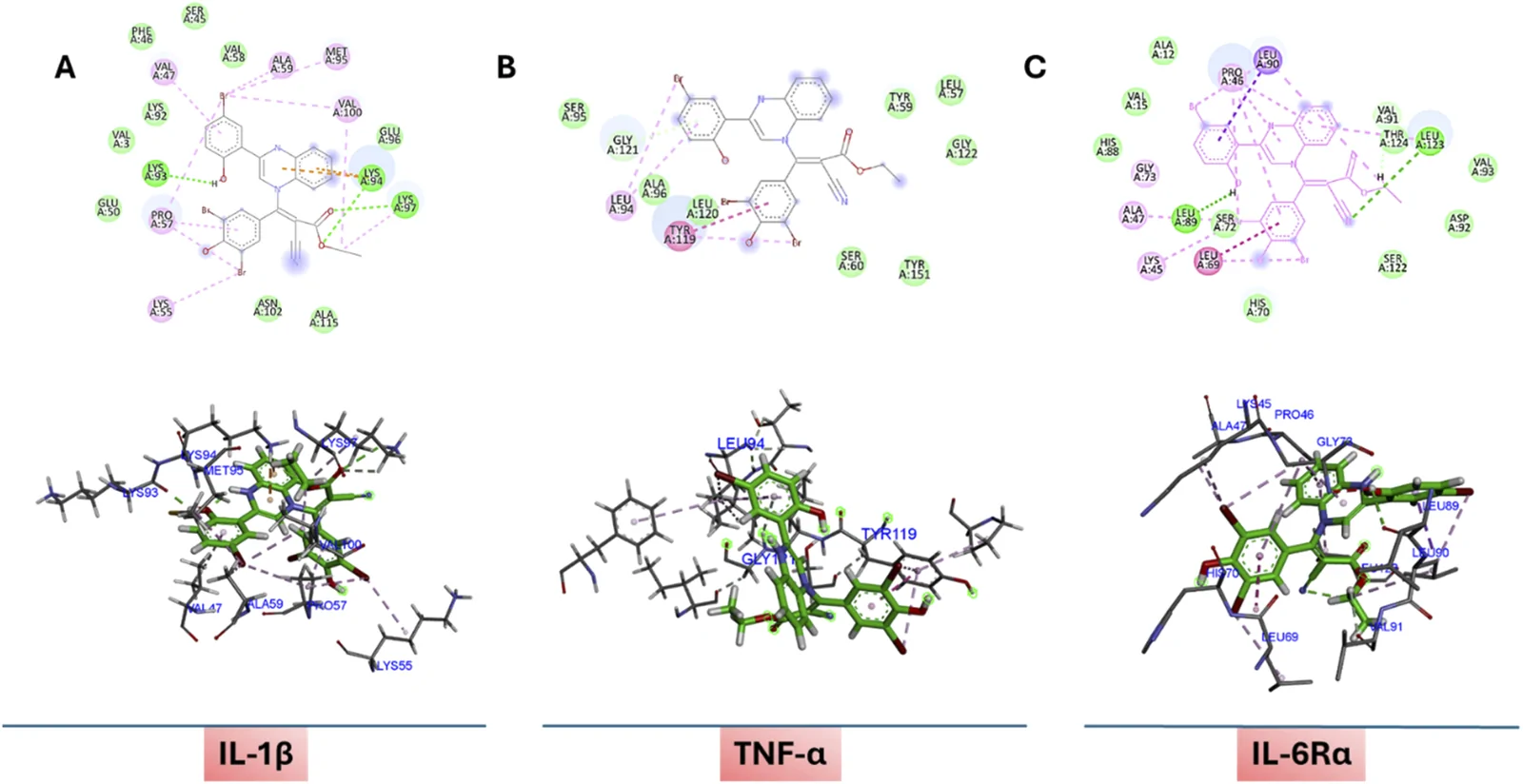
Molecular docking of compound 9 with inflammatory targets. (A) IL-1β showing hydrogen bonding and cation–π interactions with Lys94 and Lys97. (B) TNF-α illustrating π–π stacking interaction with Tyr119. (C) IL-6Rα displaying multiple stabilizing interactions, including hydrogen bonds with Leu123 and Leu89 and hydrophobic contacts with Leu90 and Leu69. Upper panels represent 2D interaction maps, while lower panels show 3D binding conformations.

Following validation, compound 9 was docked against all three targets, and the resulting binding affinities and interaction profiles are summarized in Table 1. The compound exhibited differential binding across the studied proteins, with the highest affinity observed for IL-6Rα (−7.93 kcal mol^−1^), followed by IL-1β (−6.31 kcal mol^−1^), and TNF-α (−5.73 kcal mol^−1^), suggesting a degree of target selectivity. Within the IL-1β binding site, compound 9 established key interactions with Lys94 and Lys97, including hydrogen bonding and cation–π interactions (Fig. 5A). These residues form part of a positively charged region that is also exploited by the co-crystallized ligand. Nevertheless, the native ligand exhibited an extensive interaction network involving additional residues such as Glu50, Met95, and Asn102, as well as multiple electrostatic interactions. The comparatively reduced interaction profile of compound 9 likely contributes to its lower binding affinity relative to the reference ligand (−6.31 *vs.* −9.46 kcal mol^−1^), indicating that further optimization could enhance binding complementarity within this site. Docking against TNF-α revealed that compound 9 primarily interacts with Tyr119 through π–π stacking (Fig. 5B), consistent with the binding mode of the co-crystallized inhibitor. This residue represents a key anchoring point within the hydrophobic binding pocket. While compound 9 successfully reproduces this interaction, the absence of additional stabilizing contacts, such as π–cation interactions observed in the reference ligand, may account for its slightly reduced binding energy (−5.73 *vs.* −6.13 kcal mol^−1^). In contrast, compound 9 exhibited its most favorable interaction profile with IL-6Rα, achieving a binding free energy of −7.93 kcal mol^−1^, which is notably higher than that of the reference antagonist (−6.19 kcal mol^−1^). The compound formed multiple stabilizing interactions within the receptor interface, including hydrogen bonding with Leu123 and Leu89, π–sigma interactions with Leu90, and amide–π stacking with Leu69 (Fig. 5C). Compared to the reference compound, which engaged a more limited set of residues, compound 9 exhibited a broader and more favorable interaction pattern. This enhanced binding profile suggests improved complementarity with the IL-6 receptor binding region. Overall, compound 9 displayed favorable binding interactions across all investigated targets, supported by a validated docking protocol. Its ability to engage key residues within IL-1β, TNF-α, and IL-6Rα highlights its potential as a multi-target anti-inflammatory agent. These computational findings complement the experimental data and provide a mechanistic rationale for the observed biological effects, supporting further optimization of this scaffold.

#### 2.6.2 Molecular dynamics simulation study

**Fig. 6:**
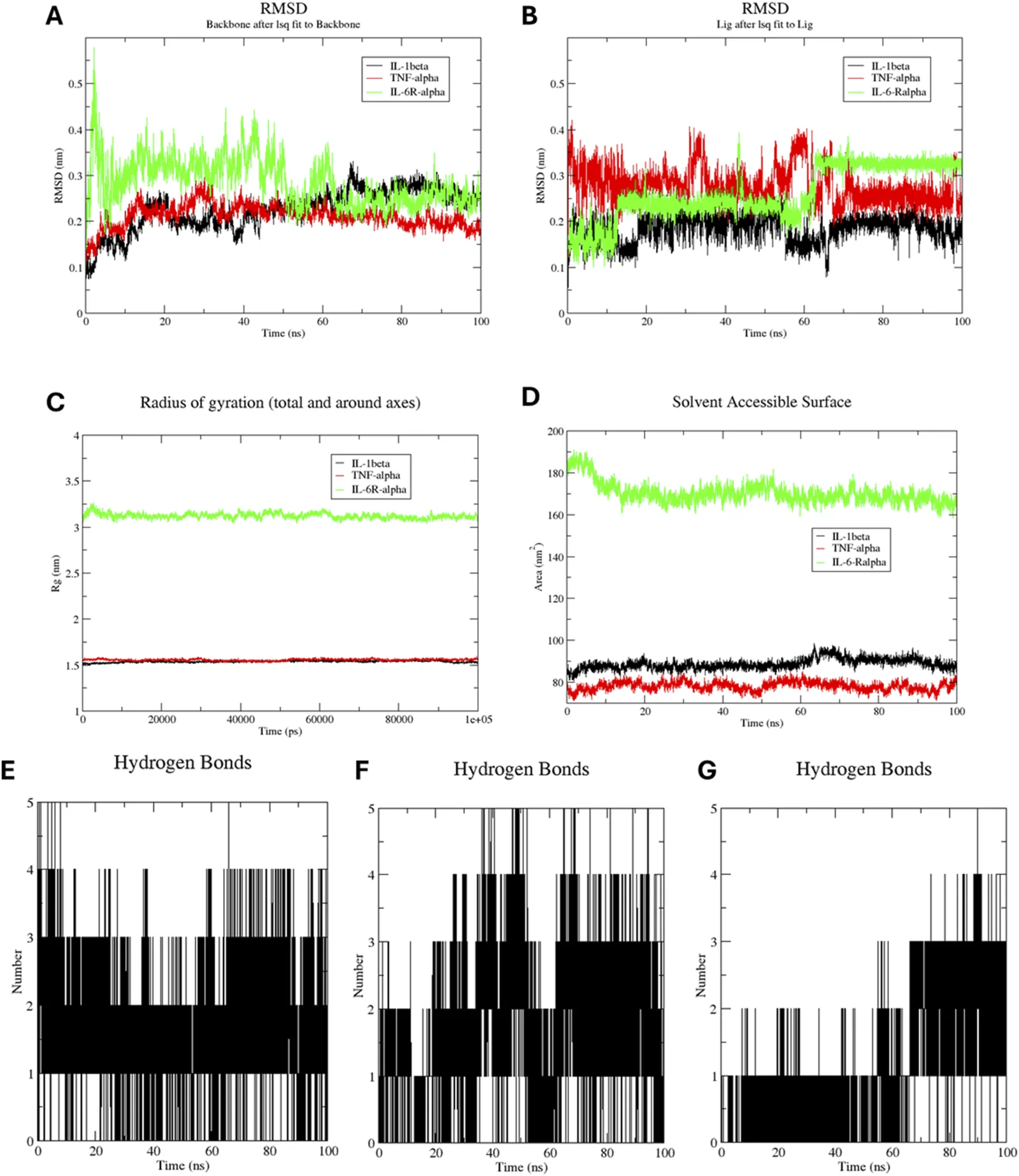
Molecular dynamics simulation analysis of compound 9 in complex with IL-1β, TNF-α, and IL-6Rα over a 100 ns trajectory. (A) Root-mean-square deviation (RMSD) of protein backbone (Cα atoms); (B) RMSD of ligand heavy atoms relative to the initial docked conformation; (C) radius of gyration (*R*_g_) of the protein complexes; (D) solvent-accessible surface area (SASA) of the proteins; (E–G) number of hydrogen bonds formed between compound 9 and IL-1β (E), TNF-α (F), and IL-6Rα (G), respectively.

The dynamic stability of the protein–ligand complexes was evaluated through 100 ns molecular dynamics simulations, with particular emphasis on structural stability, ligand retention, and interaction persistence (Fig. 6A–G). The root-mean-square deviation (RMSD) of the protein backbone (Cα atoms) was monitored to assess the overall structural integrity of the complexes (Fig. 6A). All three systems reached equilibrium within the initial 15–25 ns, indicating rapid relaxation and stable convergence of the simulations. The compound 9–IL-1β complex exhibited minimal fluctuations, stabilizing within the range of 0.17–0.27 nm after approximately 20 ns, reflecting the intrinsic rigidity of the IL-1β β-trefoil structure. Similarly, the compound 9–TNF-α complex displayed a stable RMSD profile (0.20–0.25 nm), consistent with the compact trimeric architecture of TNF-α. In contrast, the compound 9–IL-6Rα complex reached equilibrium more rapidly (<10 ns) but showed slightly higher fluctuations during the early phase (0.20–0.40 nm), followed by stabilization in the range of 0.23–0.26 nm during the final 40 ns. This behavior suggests initial conformational adjustment of the receptor–ligand interface, followed by formation of a stable binding configuration. Importantly, all RMSD values remained well below the commonly accepted threshold of ∼0.3 nm for stable protein–ligand systems, confirming that no major conformational distortions or unfolding events occurred during the simulations. To further validate the stability of the docking poses, ligand RMSD values were calculated relative to the initial conformations (Fig. 6B).

Compound 9 in the IL-1β complex exhibited very limited displacement (0.10–0.25 nm), indicating strong retention within the binding pocket. In the TNF-α complex, slightly higher fluctuations (0.20–0.40 nm) were observed, which can be attributed to the predominance of hydrophobic and π–π interactions with Tyr119, allowing a degree of conformational flexibility without ligand dissociation. Notably, the IL-6Rα complex showed the most consistent ligand stability, particularly in the equilibrated phase (0.20–0.33 nm), reflecting the presence of multiple stabilizing interactions, including hydrogen bonding and hydrophobic contacts with residues such as Leu69, Leu89, Leu90, and Leu123. These findings indicate that compound 9 maintains a stable binding orientation across all targets, with enhanced stabilization observed in IL-6Rα. The radius of gyration (*R*_g_) was analyzed to evaluate the global compactness of the proteins during simulation (Fig. 6C). All systems displayed highly stable *R*_g_ profiles with minimal fluctuations (<0.1 nm), indicating preservation of structural integrity. The mean *R*_g_ values were approximately 1.5 nm for IL-1β, 1.6 nm for TNF-α, and 3.4 nm for IL-6Rα. The absence of significant deviations in *R*_g_ reveals that compound 9 binding does not induce protein unfolding or destabilization, supporting the structural compatibility of the ligand with the target proteins. The solvent-accessible surface area (SASA) was monitored to assess changes in protein surface exposure (Fig. 6D). All complexes exhibited stable SASA profiles throughout the simulation, with mean values of approximately 80–95 nm^2^ (IL-1β), 70–80 nm^2^ (TNF-α), and 160–180 nm^2^ (IL-6Rα). The relatively lower SASA observed for TNF-α suggests partial burial of surface regions upon ligand binding, consistent with occupation of a hydrophobic binding pocket. Overall, the stable SASA profiles indicate that ligand binding does not induce significant conformational rearrangements or exposure changes in the protein structures.

Hydrogen bond analysis provided further insight into the stability of key protein–ligand interactions (Fig. 6E–G). In the IL-1β complex (Fig. 6E), hydrogen bonds with Lys94 and Lys97 were highly persistent, with occupancies exceeding 70%, along with the intermittent formation of additional stabilizing hydrogen bonds. For the IL-6Rα system (Fig. 6G), hydrogen bonding interactions with Leu89 and Leu123 also showed high occupancy (>70%) during the equilibrated phase, with occasional formation of a third hydrogen bond, reinforcing ligand stabilization at the receptor interface. In the TNF-α complex (Fig. 6F), the dominant interaction was π–π stacking with Tyr119, which remained stable throughout the trajectory. In addition, the presence of transient hydrogen bonds contributed to overall ligand stabilization despite the more hydrophobic nature of the binding site. These results indicate that the key interactions predicted by molecular docking are maintained dynamically, confirming the reliability of the docking poses and the stability of the ligand within the binding sites. Collectively, the MD simulation results indicated that compound 9 forms stable and well-equilibrated complexes with all three targets. The combination of low RMSD values, stable *R*_g_ and SASA profiles, and persistent intermolecular interactions supports the structural integrity of the complexes throughout the simulation period. Among the studied systems, the IL-6Rα complex exhibited the most favorable stability profile, characterized by rapid equilibration, consistent ligand positioning, and a robust hydrogen bonding network. These findings are in agreement with the docking results, which suggested stronger binding affinity toward IL-6Rα, and further support the potential of compound 9 as a multi-target modulator of inflammation-associated pathways.

## 3. Materials and methods

### 3.1. Chemistry

#### 3.1.1 General techniques

All spectroscopic and analytical measurements were carried out using standard laboratory instrumentation under ambient conditions unless otherwise specified. Nuclear magnetic resonance (^1^H and ^13^C NMR) spectra were recorded on a Bruker AV-400 spectrometer operating at 400 MHz for ^1^H and 100 MHz for ^13^C nuclei. Chemical shifts (*δ*) are reported in parts per million (ppm) and referenced to the residual signals of the corresponding deuterated solvents, while coupling constants (*J*) are expressed in hertz (Hz). Infrared (IR) spectra were recorded on a Shimadzu FT-IR 470 spectrophotometer using potassium bromide (KBr) discs, and characteristic absorption bands are reported in cm^−1^. Mass spectra were obtained on a Finnigan MAT SSQ-7000 mass spectrometer operating under electron ionization (EI) conditions at 70 eV, and the reported *m*/*z* values correspond to the molecular ion peaks and major fragments. Elemental (CHN) analyses were performed using a PerkinElmer 2400 series analyzer, and the experimental values were within ±0.4% of the theoretical values, confirming the purity of the synthesized compounds. Melting points were determined using a digital electrothermal melting point apparatus (Electrothermal 200) and are reported uncorrected. The progress of reactions and purity of intermediates and final compounds were monitored by thin-layer chromatography (TLC) using pre-coated silica gel plates (silica gel 60 F254), with visualization achieved under ultraviolet (UV) light. All reagents and starting materials were obtained from commercial suppliers, including Sigma-Aldrich, TCI, and Alfa Aesar, and were used as received unless otherwise indicated. Solvents were purified and dried according to standard laboratory procedures prior to use. Compounds 2a,b, 3, and 5 were synthesized according to our previously reported procedures.^21,31^

#### 3.1.2 Synthetic procedure and characterization

##### 3.1.2.1 Synthesis of 2-(5-bromo-2-hydroxyphenyl)-1,4-dihydroquinoxaline (6)

A solution of 5-bromo-2-hydroxyphenacyl bromide 5 (3.2 g, 11 mmol) in ethanol (100 mL) was treated with *o*-phenylendiamin (1.3 g, 12 mmol), followed by sodium acetate (2.05 g, 24.8 mmol). The resulting reaction mixture was allowed to heat under reflux at 78 °C, followed by TLC analysis. After being stirred under the same conditions for 12 h, the TLC analysis showed a complete reaction. The reaction was subsequently quenched by cold water and stirred for an additional 1 h. The obtained solid was filtered, washed with water, and dried under vacuum. The crude product was purified by recrystallization using a minimum amount of hot ethanol (∼60 mL) to afford compound 6 as an orange powder. Yield 67%, m.p. 212 °C. IR (KBr) *υ*_max_ (cm^−1^): 3361 (Br. OH), 3210, 3200 (2NH), 1605, 1588 (C=C), 1078 (C–O). ^1^H NMR (400 MHz, DMSO-*d*_6_, ppm) *δ*: 4.40 (s, 1H, CH-quinoxaline), 7.02–8.41 (m, 7H, Ar–H), 9.68 (s, 1H, OH), 12.20, 12.83 (s, 2H, 2NH). ^13^C NMR (100 MHz, DMSO-*d*_6_, ppm) *δ*: 159.04, 152.26 (C–O and CN), 136.77, 135.07, 133.49, 132.06, 131.92, 130.74, 128.59, 123.02, 120.03, 119.97, 118.02 (C-aromatic), 91.20 (CH-quinoxaline). MS analysis (*m*/*z*, %) = 302 (M). Anal. calcd. for C_14_H_11_BrN_2_O (Mw = 302): C, 55.63; H, 3.64; N, 9.27. Found: C, 55.48; H, 3.46; N, 9.09.

##### 3.1.2.2 Synthesis of ethyl-3-(4-hydroxyphenyl)-3-[3-(5-bromo-2-hydroxyphenyl)-1,4-dihydro-qunoxalin-1-yl]-2-cyanoacrylate (7)

A solution of compound 2a (0.45 g, 2.1 mmol) in dry ethanol (20 mL) was treated with potassium carbonate (0.725 g, 5.25 mmol). The reaction was allowed to heat under reflux at 78 °C for 3 h, before it was treated with a solution of compound 6 (0.79 g, 2.6 mmol) in dry ethanol (mL). The resulting mixture was stirred under reflux at 78 °C for an additional 18 h, as judged by TLC analysis. The reaction was cooled to 0 °C, quenched with water, and subsequently diluted with ethylacetate. The obtained mixture was washed with 1 M HCl and brine, dried over anhyd. MgSO_4_, and filtered. After being concentrated under reduced pressure, the obtained solid was purified by recrystallization using a minimum amount of hot ethanol (∼10 mL) to afford compound 7 as a pale-yellow powder. Yield 63%, mp 134 °C. IR (KBr) *υ*_max_ (cm ^−1^): 3405–3380 (br. OH), 3222 (NH), 2221 (C≡N), 1745 (C=O), 1608, 1591 (C=C), 1117, 1083, 1032 (C–O). ^1^H NMR (400 MHz, DMSO-*d*_6_, ppm) *δ*: 1.34 (t, 3H, CH_3_), 4.30 (q, 2H, OCH_2_), 5.31 (s, 1H, CH-quinoxaline), 6.96–8.31 (m, 11H, Ar–H), 8.94 (s, 1H, OH), 10.85 (s, 1H, OH), 12.94 (s, 1H, NH). ^13^C NMR (100 MHz, DMSO-*d*_6_, ppm) *δ*: 163.09 (C=O of ester), 160.33, 159.95, 158.85 (2C–O and C–N), 138.95, 136.15, 135.66, 134.46, 133.96, 132.89, 131.62, 130.93, 128.92, 126.50, 125.61, 124.49, 123.19, 122.54, 120.38, 119.96, 115.69 (C-aromatic), 110.37 (C≡N), 97.53 (CH-quinoxaline), 62.43 (C-olefinic), 58.36 (OCH_2_), 14.57 (CH_3_). MS analysis (*m*/*z*) = 517 (M). Anal. calcd. for C_26_H_20_BrN_3_O_4_ (Mw = 517): C, 60.35; H, 3.87; N, 8.12. Found: C, 60.11; H, 3.61; N, 8.02.

##### 3.1.2.3 Synthesis of ethyl-3-(3-methoxy-4-hydroxyphenyl)-3-[3-(5-bromo-2-hydroxyphenyl)-1,4-dihydroquinoxalin-1-yl]-2-cyanoacrylate (8)

Compound 2b (0.35 g, 1.4 mmol) was dissolved in dry ethanol (15 mL), and the resulting solution was treated with potassium carbonate (0.43 g, 3.2 mmol). The resulting solution was heated under reflux at 78 °C for 2 h, and subsequently, a solution of compound 6 (0.53 g, 1.8 mmol) in ethanol (10 mL) was added. The obtained mixture was allowed to heat at the same conditions for an additional 12 h. The reaction was successfully quenched with water, and the mixture was partitioned between ethylacetate and 1 M HCl solution. The organic layer was separated, and the aqueous solution was further extracted. The combined organic layers were washed with brine, dried over anhyd. MgSO_4_, filtered, and concentrated under reduced pressure to afford a solid crude product. Purification of the obtained solid by recrystallization using a minimum amount of hot ethanol (∼10 mL) as a solvent furnished compound 8 as yellow crystals. Yield 71%, mp 144 °C. IR (KBr) *υ*_max_ (cm ^−1^): 3401–3385 (br. OH), 3217 (NH), 2220 (C≡N), 1742 (C=O), 1613, 1605, 1587 (C=C), 1121, 1098, 1023 (C–O). ^1^H NMR (400 MHz, DMSO-*d*_6_, ppm) *δ*: 1.11–1.32 (t, 3H, *J* = 7.20 Hz, CH_3_), 3.83 (s, 3H, OCH_3_), 4.14–4.32 (q, 2H, *J* = 7.20 Hz, OCH_2_), 5.40 (s, 1H, CH-quinoxaline), 6.90–8.20 (m, 10H, Ar–H), 8.92 (s, 1H, OH), 10.56 (s, 1H, OH), 12.95 (s, 1H, NH). ^13^C NMR (100 MHz, DMSO-*d*_6_, ppm): 162.69 (C=O of ester), 160.33, 159.99, 157.60, 155.87 (3CO and C–N), 138.94, 136.14, 134.48, 133.60, 131.63, 130.96, 128.91, 127.61, 125.61, 124.49, 123.32, 122.57, 121.93, 120.17, 119.95, 118.84 (C-aromatic), 110.28 (C≡N), 97.52 (CH-quinoxaline), 70.43 (C-olefinic), 62.42 (OCH_2_), 56.06 (OCH_3_), 14.53 (CH_3_). MS analysis (*m*/*z*) = 548 (M + 1)^+^. Anal. calcd. for C_27_H_22_BrN_3_O_5_ (Mw = 547): C, 59.25; H, 4.02; N, 7.68. Found: C, 59.01; H, 3.88; N, 7.47.

##### 3.1.2.4 Synthesis of ethyl-3-(3,5-dibromo-4-hydroxyphenyl)-3-[3-(5-bromo-2-hydroxyphenyl)-1,4-dihydroquinoxalm-1-yl]-2-cyanoacrylate (9)

To a solution of compound 3 (1.1 g, 2.95 mmol) in anhydrous ethanol (30 mL) was added potassium carbonate (1 g, 7.4 mmol). The reaction mixture was heated at 78 °C for 2 h, and then a solution of compound 6 (1.2 g, 3.8 mmol) in anhydrous ethanol (35 mL) was added. The resulting mixture was allowed to heat under the same conditions, and the reaction progress was followed by TLC analysis. After being stirred for 16 h, the TLC indicated a complete reaction. The mixture was then cooled in ice and subsequently quenched with water. The obtained mixture was extracted with ethylacetate, acidified with 1 M HCl, and washed with brine. After being dried over anhyd. MgSO_4_, the solution was filtered and concentrated under vacuum to afford a crude solid. Purification of crude product by recrystallization using a minimum amount of hot ethanol (∼25 mL) to provide compound 9 as pale yellow crystals. Yield 67%, mp 166 °C. IR (KBr) *υ*_max_ (cm ^−1^): 3405–3383 (br. OH), 3225 (NH), 2225 (CN), 1748 (C=O), 1611, 1603, 1541 (C=C), 1121, 1088, 1023 (C–O). ^1^HNMR (400 MHz, DMSO-*d*_6_, ppm) *δ*: 1.18–1.31 (t, 3H, CH_3_), 4.07–4.55 (q, 2H, OCH_2_), 5.60 (s, 1H, CH-quinoxaline), 7.23–8.30 (m, 9H, Ar–H), 8.92 (s, 1H, OH), 10.21 (s, 1H, OH), 11.73 (s, 1H, NH). ^13^C NMR (100 MHz, DMSO-*d*_6_, ppm) *δ*: 163.11, 162.68 (C=O of ester) 160.31, 159.95, 157.60 (2CO and C–N), 148.30, 142.49, 138.94, 136.14, 134.49, 133.72, 131.05, 128.92, 127.61, 124.50, 123.34, 121.92, 120.38, 119.95, 117.68, 116.51, 115.85, 114.62 (C-aromatic), 110.40 (C≡N), 97.58 (CH-quinoxaline), 62.42 (C-olefinic), 56.09 (OCH_2_), 14.53 (CH_3_). Anal. calcd. for C_26_H_18_Br_3_N_3_O_4_ (Mw = 673): C, 46.36; H, 2.67; N, 6.24. Found: C, 46.16; H, 2.52; N, 6.05.

##### 3.1.2.5 Synthesis of 2-(5-bromo-2-acetoxyphenyl)-1,4-diacetylquinoxaline (10)

Compound 9 (0.9 g, 1.34 mmol) was dissolved in acetic anhydride (10 mL), and the resulting solution was allowed to heat under reflux at 140 °C for 12 h. The obtained mixture was then cooled to room temperature and subsequently quenched by pouring into ice-H_2_O. The mixture was allowed to stir under the same conditions for 1 h, and kept at ambient temperature for an additional 16 h. The obtained solid was collected by filtration, washed with water, and dried under vacuum. The crude product was purified by recrystallization from a minimum amount of hot ethanol (∼15 mL) to provide compound 10 as colorless crystals. Yield 52%, mp 205 °C. IR (KBr) *υ*_max_ (cm^−1^): 1752, 1705, 1693 (3C=O), 1583 (C=C), 1063 (C–O). ^1^H NMR (400 MHz, DMSO-*d*_6_, ppm) *δ*: 1.80 (s, 6H, 2COCH_3_), 2.11 (s, 3H, COCH_3_), 5.30 (s, 1H, CH-quinoxaline), 6.70–7.78 (m, 7H, Ar–H). ^13^C NMR (100 MHz, DMSO-*d*_6_, ppm) *δ*: 173.54, 172.06, 167.87 (3C=O), 158.48, 155.69, 151.35 (C–O and 2C–N), 137.90, 134.80, 134.13, 129.15, 124.30, 123.11, 120.14, 115.85, 115.43, 115.20 (C-aromatic and olefinic), 109.80 (CH-quinoxaline), 24.06 (CH_3_), 23.76 (CH_3_), 22.97 (CH_3_). MS analysis (*m*/*z*) = 428 (M). Anal. calcd. for C_20_H_17_BrN_2_O_4_ (Mw = 428): C, 56.34; H, 3.52; N, 6.572. Found: C, 56.13; H, 3.33; N, 6.363. The filtrate was concentrated and left for 18 h at ambient temperature to provide a solid, which was isolated by filtration, washed with water, and dried. Recrystallization of crude solid from a minimum amount of hot benzene (∼8 mL) yielded compound 11 (ethyl-3-(3,5-dibromo-4-acetoxyphenyl)-2-cyanoacrylate) as colorless crystals. Yield 38%. mp 114 °C. IR (KBr) *υ*_max_ (cm^−1^): 2224 (CN), 1715 (C=O of ester), 1743 (C=O), 1605, 1582 (C=C), 1121, 1083, 1033 (C–O). ^1^H NMR (400 MHz, DMSO-*d*_6_, ppm) *δ*: 1.27–1.31 (t, 3H, *J* = 7.20 Hz, CH_3_), 1.91 (s, 3H, COCH_3_), 4.26–4.32 (q, 2H, *J* = 7.20 Hz, OCH_2_), 8.24 (s, 1H, H-olefinic), 8.29 (s, 2H, Ar–H). ^13^C NMR (100 MHz, DMSO-*d*_6_, ppm) *δ*: 172.60, 162.25 (2C=O of ester and acetoxy), 155.82 (C–O), 152.28, 151.10, 135.50, 134.54, 125.88, 116.17, 113.31 (C-aromatic and olefinic), 101.40 (CN), 62.84 (OCH_2_), 21.5 (COCH_3_), 14.43 (CH_3_). Anal. calcd. for C_14_H_11_Br_2_NO_4_ (M.wt. = 415): C, 40.48; H, 2.65; N, 3.37. Found: C, 40.36; H, 2.33; N, 3.13.

### 3.2. Screening of cytotoxic activity

The antiproliferative activity of the synthesized quinoxaline derivatives (6–10) was evaluated against a panel of human cancer cell lines (obtained from the Regional Center for Mycology and Biotechnology at Al-Azhar University, Egypt), including HepG-2 (hepatoblastoma), HCT-116 (colon carcinoma), MCF-7 (breast adenocarcinoma), and Panc-1 (pancreatic carcinoma), in addition to the normal human lung fibroblast cell line WI-38, using the MTT colorimetric assay.^30,57^ The cytotoxic effect of the compounds was compared with the reference anticancer drug doxorubicin. Briefly, cells were cultured in appropriate growth medium supplemented with 10% fetal bovine serum and maintained at 37 °C in a humidified incubator with 5% CO_2_. For the assay, cells were seeded into 96-well plates (1 × 10^5^ cells per well) and allowed to adhere for 24 h. Following attachment, cells were treated with increasing concentrations of compounds 6–10, as well as doxorubicin. Each concentration was tested in triplicate. Treated cells were incubated for 24 h under standard culture conditions. After the treatment period, cell viability was assessed by adding MTT solution (0.5 mg mL^−1^ final concentration) to each well, followed by incubation for 4 h at 37 °C. Viable cells reduced the yellow tetrazolium salt to insoluble purple formazan crystals. The culture medium was then carefully removed, and the formed crystals were dissolved in DMSO with gentle shaking to ensure complete solubilization. The absorbance was measured at 570 nm using a microplate reader, with background correction where applicable. Cell viability was calculated as a percentage relative to untreated control cells. Dose–response curves were constructed by plotting cell viability against compound concentration, and IC_50_ values (µM) were determined using non-linear regression analysis. All experiments were performed in triplicate, and results were expressed as mean ± SD. The incubation period was fixed at 24 h for all tested compounds and the reference drug doxorubicin. Since doxorubicin exhibits marked time-dependent cytotoxicity, IC_50_ values reported in the present study should be interpreted within the context of this exposure period.^32–35^

### 3.3. Cell cycle analysis by flow cytometry

The effect of compound 9 on cell cycle distribution was evaluated in HepG2 cells using propidium iodide-based DNA content analysis by flow cytometry.^23^ HepG2 cells were seeded in 6-well plates (2 × 10^5^ cells per well) and allowed to attach overnight under standard culture conditions (37 °C, 5% CO_2_). Cells were then treated with compound 9 at its IC_50_ concentration for 24 h, while untreated cells served as the control group. Following treatment, cells were harvested by trypsinization, collected by centrifugation, and washed twice with cold PBS. The cell pellets were then fixed by gradual addition of ice-cold ethanol (70%) while vortexing and stored at −20 °C for at least 2 h to ensure proper fixation and permeabilization. After fixation, cells were washed with PBS to remove residual ethanol and subsequently resuspended in a propidium iodide/RNase staining solution. The samples were incubated at 37 °C for 30 min in the dark to allow complete RNA degradation and uniform DNA staining. Cell cycle distribution was analyzed using a flow cytometer equipped with a 488 nm excitation laser, and PI fluorescence was detected in the appropriate emission channel. The proportions of cells in G0/G1, S, and G2/M phases were determined using dedicated cell cycle analysis software.

### 3.4. Apoptosis analysis by Annexin V-FITC/PI staining

The apoptotic effect of compound 9 was evaluated in HepG2 cells using Annexin V-FITC/propidium iodide dual staining followed by flow cytometric analysis.^58^ HepG2 cells were seeded in 6-well plates (2 × 10^5^ cells per well) and allowed to adhere overnight under standard culture conditions (37 °C, 5% CO_2_). Cells were then treated with compound 9 at its IC_50_ concentration for 24 h, while untreated cells served as the control. After treatment, cells were harvested by trypsinization, collected by centrifugation, and washed twice with cold PBS to remove residual medium. The cell pellets were then gently resuspended in Annexin V binding buffer. For staining, Annexin V-FITC (5 µL) and propidium iodide (PI, 5 µL) were added to each sample, followed by incubation at room temperature for 15 min in the dark. After staining, samples were immediately analyzed by flow cytometry. Fluorescence signals were detected using appropriate channels, with Annexin V-FITC measured in the FITC channel (FL1) and PI in the PE channel (FL2). Cell populations were discriminated into four quadrants: viable cells (Annexin V^−^/PI^−^), early apoptotic cells (Annexin V^+^/PI^−^), late apoptotic cells (Annexin V^+^/PI^+^), and necrotic cells (Annexin V^−^/PI^+^). Data acquisition and analysis were performed using dedicated flow cytometry software to quantify the percentage of cells in each population.

### 3.5. Assessment of apoptosis-related genes in HepG2 cells

The expression levels of key apoptosis-related genes were evaluated in HepG2 cells following treatment with compound 9 using quantitative real-time PCR (qRT-PCR).^59,60^ HepG2 cells were seeded (1 × 10^5^ cells per mL) in 6-well plates and incubated overnight under standard culture conditions (37 °C, 5% CO_2_). Cells were then treated with compound 9 at its IC_50_ concentration for 24 h, while untreated cells served as the negative control, and doxorubicin was used as a reference treatment under identical conditions. Following treatment, total RNA was extracted using a commercial RNA isolation kit (RNeasy Mini Kit, Qiagen, Germany) according to the manufacturer's instructions. The concentration and purity of RNA were assessed spectrophotometrically. Equal amounts of purified RNA were reverse-transcribed into cDNA using a standard reverse transcription kit. Quantitative real-time PCR was performed using a SYBR Green-based master mix in a total reaction volume of 25 µL, containing master mix, gene-specific primers, cDNA template, and nuclease-free water. The expression levels of p53, BAX, Bcl-2, cytochrome c (CYC), and caspase-7 were analyzed, with GAPDH used as the internal reference gene (Table S1). PCR amplification was carried out under the following conditions: initial activation at 95 °C for 10 min, followed by 40 cycles of denaturation at 95 °C for 15 s and annealing/extension at 60 °C for 30 s. Relative gene expression levels were calculated using the 2^−ΔΔ*C*_*t*_^ method after normalization to GAPDH and expressed as fold change relative to untreated control cells. All experiments were conducted in triplicate, and data were presented as mean ± SD.

### 3.6. Assessment of macrophage RAW 264.7 cell viability

The cytocompatibility of compound 9 was evaluated in murine macrophage RAW 264.7 cells (obtained from the Regional Center for Mycology and Biotechnology at Al-Azhar University, Egypt) using the MTT assay, both in the absence and presence of inflammatory stimulation.^59,61^ RAW 264.7 cells were cultured in DMEM supplemented with 10% fetal bovine serum and maintained at 37 °C in a humidified atmosphere containing 5% CO_2_. Cells were seeded into 96-well plates (1 × 10^4^ cells per well) and allowed to adhere overnight. Cells were then treated with compound 9 at various concentrations (12.5, 25, 50, 100, 150, and 200 µM) in the absence or presence of lipopolysaccharide (LPS). For inflammatory conditions, cells were pretreated with compound 9 for 2 h, followed by stimulation with LPS (1 µg mL^−1^) and incubation for an additional 24 h. Control groups included untreated cells and LPS-stimulated cells without compound treatment. Following the treatment period, MTT reagent (0.5 mg mL^−1^ final concentration) was added to each well and incubated for 4 h at 37 °C. The culture medium was then carefully removed, and the formed crystals were dissolved using DMSO. The absorbance was measured at 570 nm using a microplate reader, with background correction at a reference wavelength where applicable. Cell viability was expressed as a percentage relative to the corresponding control group (untreated cells for non-stimulated conditions and LPS-treated cells for stimulated conditions). All experiments were conducted in triplicate, and results were presented as mean ± SD.

### 3.7. Assessment of intracellular ROS in LPS-induced RAW 264.7 cells

Intracellular ROS levels were quantified in LPS-stimulated RAW 264.7 macrophages using a fluorometric assay to evaluate the antioxidant and anti-inflammatory activity of compound 9.^62^ The assay was performed using a commercially available ROS detection kit (Sigma-Aldrich, MAK142), following the manufacturer's instructions with minor modifications. RAW 264.7 cells were cultured in DMEM supplemented with 10% FBS and maintained at 37 °C in a humidified incubator containing 5% CO_2_. Cells were seeded into black 96-well plates at a density of approximately 1 × 10^5^ cells per well and allowed to adhere overnight. For treatment, cells were pre-incubated with compound 9 at different concentrations (2.5, 5, 10, 20, 40, and 80 µM) for 2 h in serum-free medium. Following pretreatment, inflammation was induced by adding lipopolysaccharide (LPS, 1 µg mL^−1^), and cells were incubated for an additional 24 h. Control groups included untreated cells and LPS-stimulated cells without treatment. After incubation, the culture medium was carefully removed, and cells were gently washed with PBS to eliminate residual serum and phenol red. Subsequently, cells were incubated with the ROS-sensitive fluorescent probe prepared according to the kit protocol and kept in the dark at 37 °C for 60 min. Fluorescence intensity was measured using a microplate reader at excitation/emission wavelengths of 650/675 nm. The recorded fluorescence intensity was proportional to intracellular ROS levels. Data were expressed as relative ROS production compared to the LPS-treated control group, and the percentage inhibition of ROS generation was calculated. All experiments were performed in triplicate, and data were expressed as mean ± SD.

### 3.8. Gene expression analysis of inflammatory markers in RAW 264.7 cells

The effect of compound 9 on the expression of key inflammatory mediators was evaluated in LPS-stimulated RAW 264.7 macrophages using quantitative real-time PCR (qRT-PCR).^63,64^ RAW 264.7 cells were seeded in 6-well plates (1 × 10^5^ cells per mL) and allowed to adhere overnight under standard culture conditions (37 °C, 5% CO_2_). Cells were pretreated with compound 9 at 10 µM, followed by stimulation with LPS (1 µg mL^−1^) for 24 h. Celecoxib was used at 10 µM as a reference control,^65^ while LPS-treated cells served as a positive control. Following treatment, total RNA was extracted using a commercial RNA isolation kit (RNeasy Mini Kit, Qiagen, Inc., Valencia, CA, USA) according to the manufacturer's protocol. The purity and concentration of RNA were assessed spectrophotometrically. Complementary DNA (cDNA) was synthesized from equal amounts of RNA using a reverse transcription kit (RT^2^ First Strand Kit, Qiagen, Inc., Valencia, CA, USA). Quantitative real-time PCR was performed using a SYBR Green-based master mix (RT^2^ SYBR Green ROX FAST Master Mix, Qiagen, Inc., Valencia, CA, USA) in a total reaction volume of 25 µL, containing master mix, gene-specific primers, cDNA template, and nuclease-free water. Validated primers specific for TNF-α, IL-1β, and IL-6 were used, with GAPDH serving as the internal housekeeping gene (Table S2). The PCR amplification was carried out under the following conditions: initial activation at 95 °C for 10 min, followed by 40 cycles of denaturation at 95 °C for 15 s and annealing/extension at 60 °C for 30 s. A melting curve analysis was performed to confirm amplification specificity. Relative gene expression levels were calculated using the 2^−ΔΔ*C*_*t*_^ method after normalization to GAPDH and expressed as fold change relative to LPS-treated cells. All experiments were conducted in triplicate, and results were presented as mean ± SD.

### 3.9. Assessment of nitric oxide production in LPS-induced RAW 264.7 cells

The effect of compound 9 on NO production was assessed in LPS-stimulated RAW 264.7 macrophages using the Griess reaction, which quantifies nitrite as a stable end-product of NO metabolism.^66^ RAW 264.7 cells were seeded into 24-well plates at a density of approximately 1.5 × 10^5^ cells per well and allowed to attach overnight under standard culture conditions (37 °C, 5% CO_2_). Cells were then pretreated with compound 9 at 10 µM for 2 h prior to stimulation with LPS (1 µg mL^−1^). Celecoxib was used at 10 µM as the reference anti-inflammatory drug under the same experimental conditions,^65^ and LPS-treated cells served as the inflammatory control. After 24 h of incubation, the culture supernatants were collected for nitrite determination. For NO quantification, 50 µL of each cell culture supernatant was mixed with an equal volume of Griess reagent and incubated at room temperature for 20 min in the dark to allow color development. The absorbance was then measured at 540 nm using a microplate reader. Nitrite concentration was determined from a calibration curve prepared using sodium nitrite standard solutions. The obtained values were expressed as relative fold change compared with the LPS-treated control group. All measurements were performed in triplicate, and the results were expressed as mean ± SD.

### 3.10. Molecular docking studies

The three-dimensional structures of the selected inflammatory targets were retrieved from the RCSB Protein Data Bank. The investigated proteins included interleukin-1β (IL-1β, PDB ID: 8C3U),^54^ tumor necrosis factor-α (TNF-α, PDB ID: 2AZ5),^55^ and the extracellular domain of interleukin-6 receptor (IL-6Rα, PDB ID: 1N26).^56^ Prior to docking, protein structures were prepared using AutoDockTools by removing crystallographic water molecules and any co-crystallized ligands, followed by the addition of polar hydrogen atoms. Partial atomic charges were assigned using the Gasteiger–Marsili method, and the prepared structures were subsequently converted into PDBQT format for docking simulations. The chemical structures of compound 9 and reference ligands were constructed using ChemSketch and subjected to geometry optimization to obtain energetically stable conformations. The optimized ligands were then processed using AutoDockTools to assign appropriate torsions and charges and converted into PDBQT format for compatibility with the docking software. Molecular docking simulations were performed using the Lamarckian Genetic Algorithm implemented in AutoDock 4.2. A grid box with dimensions of 40 × 40 × 40 Å was defined to encompass the active binding region of each protein target. For IL-1β and TNF-α, the grid was centered on the coordinates of the respective co-crystallized ligands to ensure accurate targeting of the active sites. To validate the docking protocol, the native ligands were re-docked into their corresponding binding pockets, and the resulting poses were compared with the crystallographic conformations. In the case of IL-6Rα, which lacks a co-crystallized small-molecule ligand, the docking region was defined based on reported key residues involved in ligand recognition, and a previously reported antagonist was employed as a reference to support the docking setup. Docking parameters were configured to allow extensive conformational sampling, with 2.5 × 10^6^ energy evaluations per run, 100 independent docking runs, and a population size of 150 individuals. The generated docking conformations were clustered based on binding energy, and the most favorable poses within a 3 kcal mol^−1^ range of the lowest-energy conformation were selected for further evaluation. The binding interactions between ligands and target proteins were analyzed using BIOVIA Discovery Studio Visualizer.^67–69^ Both two-dimensional and three-dimensional interaction profiles were examined to identify key binding features, including hydrogen bonding, hydrophobic interactions, and electrostatic contacts, which contribute to ligand affinity and stability within the active site.

### 3.11. Molecular dynamics simulation studies

To investigate the dynamic stability and conformational behavior of the predicted protein–ligand complexes, molecular dynamics (MD) simulations were performed for 100 ns using GROMACS 2023.1.^70^ The most energetically favorable docking pose of compound 9 in complex with each target protein, IL-1β (PDB ID: 8C3U), TNF-α (PDB ID: 2AZ5), and IL-6Rα (PDB ID: 1N26), was selected as the initial configuration for the simulations. The protein topologies were generated using the CHARMM36 force field, which is widely validated for biomolecular systems. Ligand parameters for compound 9 were derived using the CHARMM General Force Field (CGenFF) *via* the online server, ensuring compatibility with the protein force field and accurate assignment of atom types, charges, and bonded parameters.^71^ Each protein–ligand complex was placed in a periodic dodecahedral simulation box, maintaining a minimum distance of 10 Å between the solute and the box edges to avoid artefacts from periodic images. The systems were solvated using the TIP3P water model, and appropriate numbers of sodium and chloride ions were added to neutralize the system and achieve an ionic strength of 0.15 M, mimicking physiological conditions. Prior to simulation, energy minimization was carried out using the steepest descent algorithm to eliminate steric clashes and optimize system geometry. The minimization was continued until the maximum force fell below 10 kJ mol^−1^ nm^−1^, with a maximum of 50 000 steps. Equilibration was subsequently performed in two stages. First, an NVT ensemble was applied for 10 ps at 310 K using the V-rescale thermostat, while positional restraints were imposed on heavy atoms to allow solvent and ions to relax around the complex.^72^ This was followed by an NPT equilibration phase for an additional 10 ps at 310 K and 1 bar pressure, employing the Parrinello–Rahman barostat to stabilize system density and volume. Production MD simulations were then conducted for 100 ns with a time step of 2 fs. Long-range electrostatic interactions were treated using the Particle Mesh Ewald (PME) method, with a cutoff of 12 Å for short-range interactions. van der Waals interactions were handled using the same cutoff with a switching function applied from 10 Å. All bonds involving hydrogen atoms were constrained using the LINCS algorithm, enabling stable integration with the selected time step. Trajectory data were recorded every 10 ps for subsequent analysis. Post-simulation analyses were performed using built-in GROMACS utilities and visualized with XMGRACE. The structural stability and behavior of each system were evaluated through multiple parameters, including root-mean-square deviation (RMSD) of protein backbone atoms and ligand coordinates, radius of gyration (*R*_g_) to assess protein compactness, solvent-accessible surface area (SASA) to monitor changes in surface exposure, and hydrogen bond analysis to determine the persistence of key protein–ligand interactions throughout the simulation.

## 4. Conclusion

In this study, a rational hybridization strategy combining the quinoxaline scaffold with an α,β-unsaturated cyanoacrylate moiety successfully yielded a new class of multifunctional compounds with promising biological profiles. The synthetic approach provided structurally diverse derivatives (6–10), enabling systematic evaluation of their pharmacological potential. Among them, compound 9 emerged as the most active analogue, exhibiting a favorable balance between potency and selectivity, particularly against hepatocellular carcinoma cells, while maintaining reduced toxicity toward normal fibroblasts. Mechanistic investigations revealed that the anticancer activity of compound 9 is primarily mediated through disruption of cell cycle progression and activation of the intrinsic apoptotic pathway, as evidenced by modulation of key regulatory genes and apoptotic markers. Importantly, the compound also exhibited pronounced anti-inflammatory activity by attenuating oxidative stress and suppressing the production of major pro-inflammatory mediators, indicating its ability to interfere with interconnected signaling networks linking inflammation and tumor progression. This dual functionality highlights the therapeutic relevance of targeting both cancer cell survival and the inflammatory microenvironment within a single molecular framework. Computational studies further supported the experimental findings, revealing that compound 9 can effectively engage multiple inflammation-related targets, including IL-1β, TNF-α, and IL-6Rα. The stability of these interactions throughout molecular dynamics simulations reinforces the proposed multi-target mode of action and provides a structural basis for the observed biological effects. Despite these encouraging results, further investigations are warranted to fully elucidate the pharmacokinetic properties, target selectivity, and *in vivo* efficacy of this compound class. Future studies should focus on structural optimization to enhance potency and selectivity, detailed mechanistic validation at the protein level, and evaluation in relevant animal models to confirm therapeutic potential. Additionally, exploration of structure–activity relationships within this hybrid scaffold may facilitate the development of next-generation analogues with improved drug-like properties. Overall, the present work establishes quinoxaline–cyanoacrylate hybrids as a promising platform for the development of multi-target agents with combined anticancer and anti-inflammatory activities, offering a valuable direction for future drug discovery efforts.

## Author contributions

Conceptualization, S. Z. A., G. A. B., H. A. M. S., A. A. A., and E. M. S.; methodology, S. Z. A., G. A. B., H. A. M. S., L. M. A. A., A. M. A., A. A. A., and E. M. S.; software, S. Z. A., G. A. B., H. A. M. S., L. M. A. A., A. M. A., A. A. A., and E. M. S.; validation, S. Z. A., G. A. B., H. A. M. S., L. M. A. A., A. M. A., A. A. A., and E. M. S.; formal analysis, S. Z. A., G. A. B., H. A. M. S., L. M. A. A., A. M. A., A. A. A., and E. M. S.; investigation, S. Z. A., G. A. B., A. M. A., A. A. A., and E. M. S.; resources, S. Z. A., G. A. B., H. A. M. S., A. A. A., and E. M. S.; data curation, S. Z. A., G. A. B., H. A. M. S., and E. M. S.; writing – original draft preparation S. Z. A., G. A. B., H. A. M. S., L. M. A. A., A. M. A., A. A. A., and E. M. S.; writing—review and editing, S. Z. A., G. A. B., H. A. M. S., L. M. A. A., A. M. A., A. A. A., and E. M. S.; visualization, S. Z. A., G. A. B., H. A. M. S., and E. M. S.; supervision, S. Z. A., A. A. A., and E. M. S.; project administration, S. Z. A., G. A. B., H. A. M. S., A. A. A., and E. M. S.; funding acquisition, S. Z. A., A. A. A., and E. M. S. All authors have read and agreed to the published version of the manuscript.

## Conflicts of interest

The authors affirm that they are not aware of any personal or financial conflicts that would have seemed to affect the findings of this study's research.

## Supplementary Material

RA-016-D6RA03332F-s001

## Data Availability

Data supporting the results reported in this manuscript are included in this article and as part of the supplementary information (SI). The raw data supporting the conclusions of this article will be made available by the authors without any undue reservation. Supplementary information is available. See DOI: https://doi.org/10.1039/d6ra03332f.
