# Supplementary material for: Quinoxaline-based cyanoacrylate hybrids as multi-target anti-proliferative and anti-inflammatory agents with potent activity against hepatocellular carcinoma: induction of apoptosis and modulation of oxidative stress signaling
Source: RSC Adv. 2026 Aug 21;16(41):49178–97. doi: 10.1039/d6ra03332f (PMC13494832; doi:10.1039/d6ra03332f)
Supplement: Supplementary file 1 [file RA-016-D6RA03332F-s001.pdf]

Supplementary Information (SI) for RSC Advances.
This journal is © The Royal Society of Chemistry 2026

Supporting Information

Table S1: List of apoptosis-related genes utilized in this study.

Gene	Forward Primer (5′→3′)	Reverse Primer (5′→3′)
**TP53 (p53)**	CAGCACATGACGGAGGTTGT	TCATCCAAATACTCCACACGC
**BAX**	CCCGAGAGGTCTTTTTCCGAG	CCAGCCCATGATGGTTCTGAT
**BCL2**	GGTGAACTGGGGGAGGATTG	GCTGGGAGGAGAAGATGC
**CYCS**	CTTTCGGGAGGCAAGCATAA	CAGGGTATCCTCTCCCCAGT
**CASP7**	GGAACAGGCTCTGGATGAAC	CACTGGCATTTGTCTTCAGG
**GAPDH**	GAAGGTGAAGGTCGGAGTCA	GAAGATGGTGATGGGATTTC

Table S2. List of Inflammatory genes utilized in this study.

Gene	Forward Primer (5′→3′)	Reverse Primer (5′→3′)
**TNF-α**	CCCTCACACTCAGATCATCTTCT	GCTACGACGTGGGCTACAG
**IL-1β**	GCAACTGTTCCTGAACTCAACT	ATCTTTTGGGGTCCGTCAACT
**IL-6**	TAGTCCTTCCTACCCCAATTTCC	TTGGTCCTTAGCCACTCCTTC
**GAPDH**	AGGTCGGTGTGAACGGATTTG	TGTAGACCATGTAGTTGAGGTCA

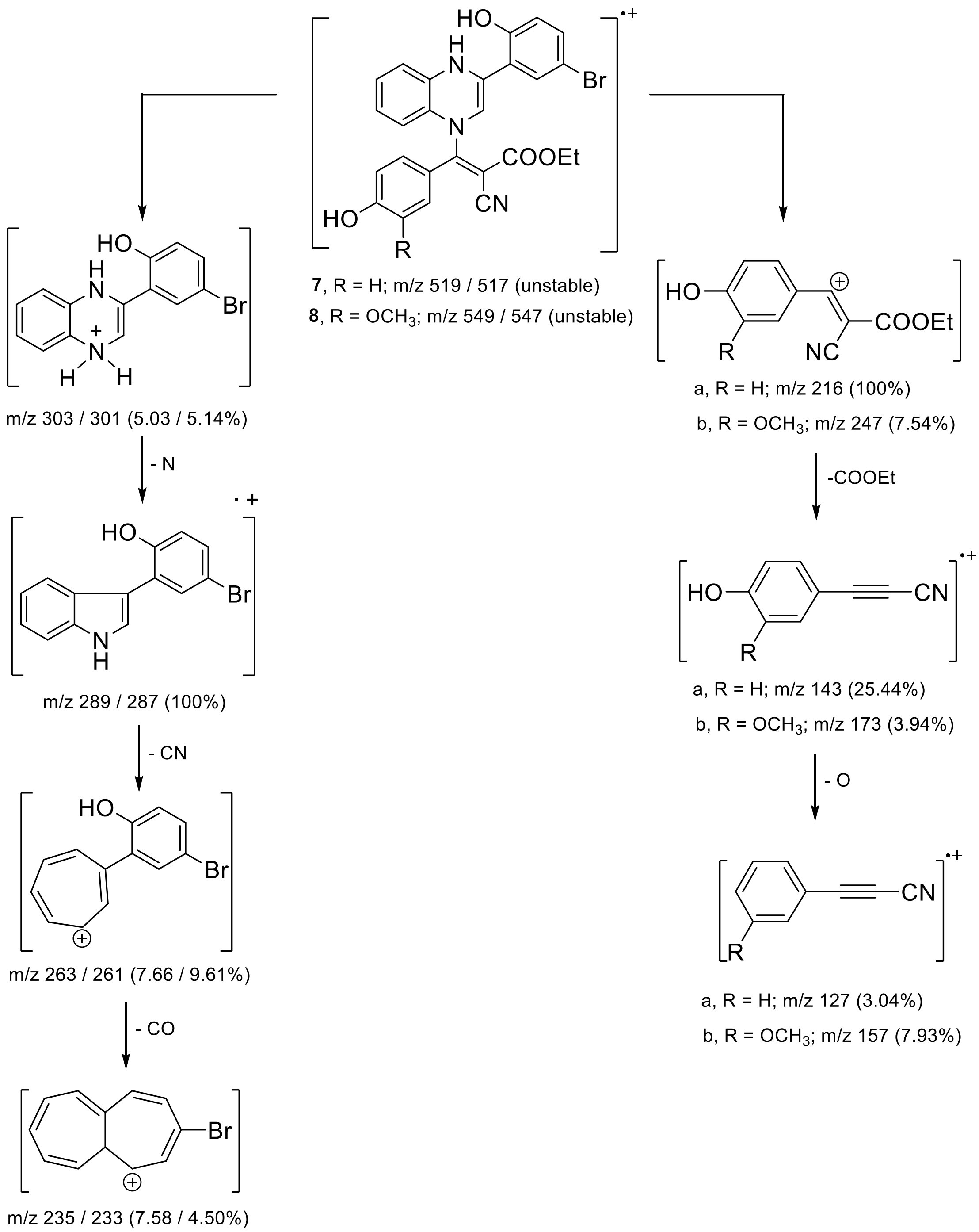

Figure S1: Mass spectrum fragmentation pattern of compounds **7** and **8**.

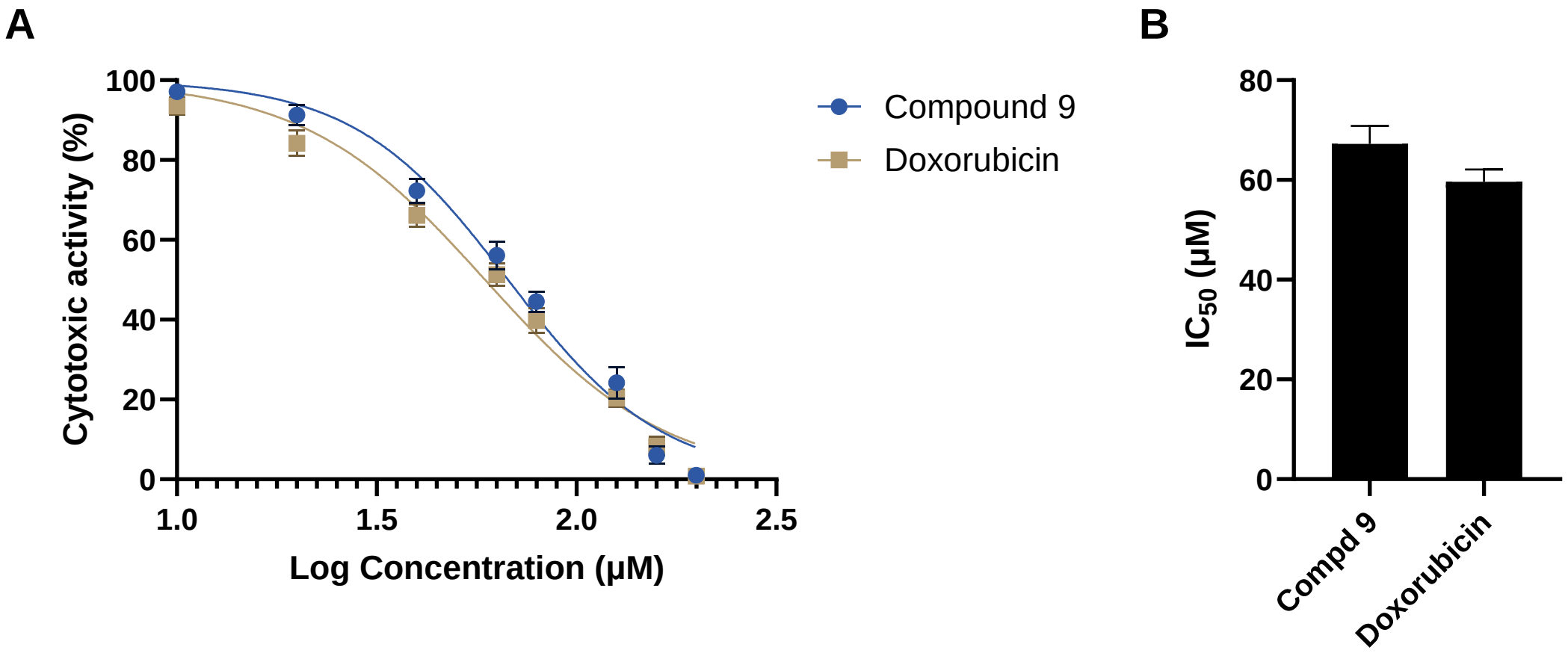

Figure S2. Cytotoxicity assessment of compound **9** and doxorubicin against WI-38 normal human fibroblast cells using the MTT assay. **(A)** Dose–response curves showing the effect of increasing concentrations (log μM) on cell viability (%). **(B)** Corresponding IC_{50} values (μM) for compound **9** and doxorubicin. Data are presented as mean ± SD

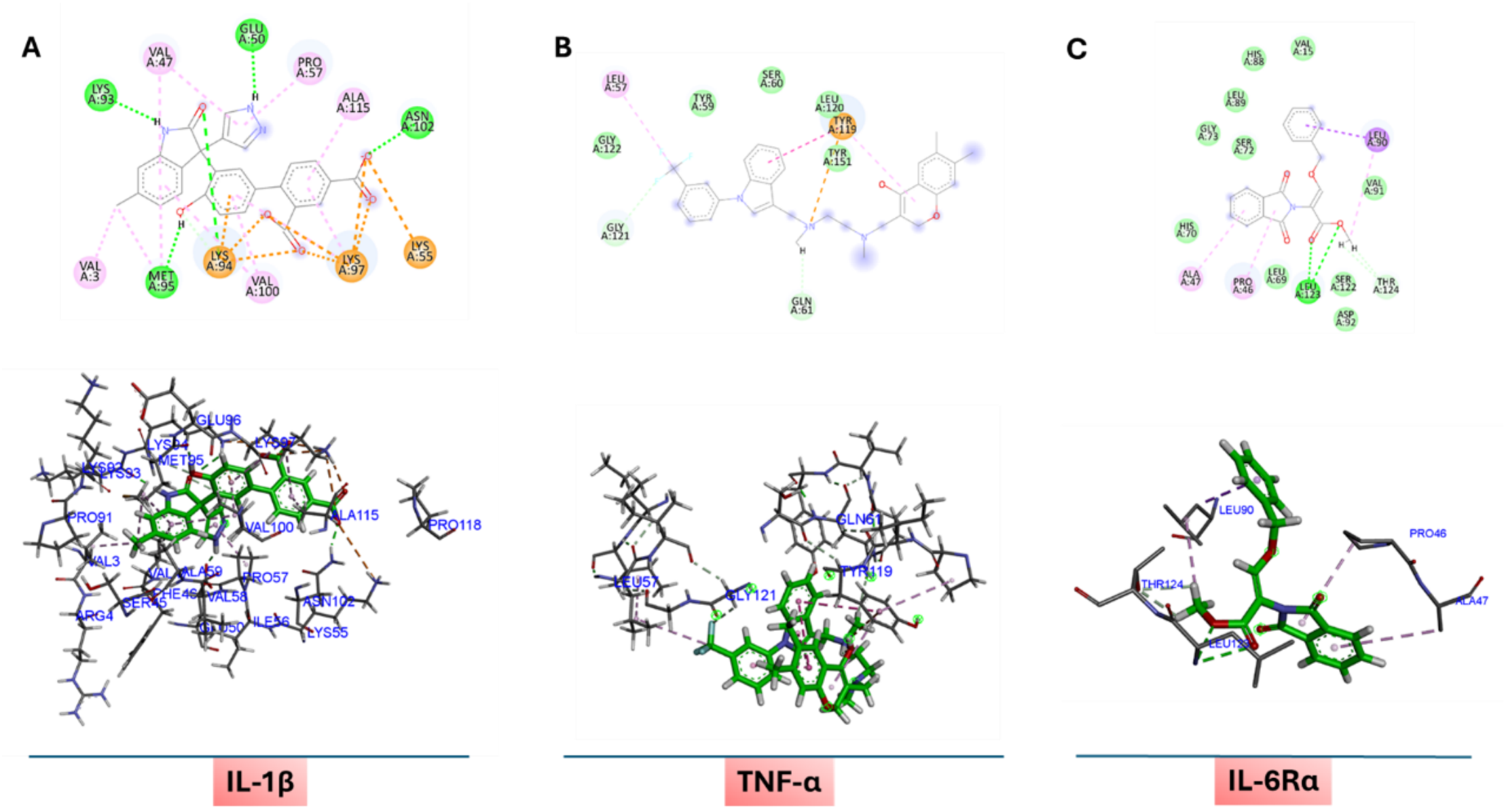

Figure S3. Molecular interactions of co-crystallized ligands within inflammatory targets. (**A**) IL-1β showing extensive hydrogen bonding and electrostatic interactions with key residues including Lys94 and Lys97. (**B**) TNF-α illustrating dominant aromatic interactions with Tyr119 within the hydrophobic pocket. (**C**) IL-6Rα displaying binding interactions with residues such as Leu123 and Leu90 at the receptor interface. Upper panels represent 2D interaction maps, while lower panels show 3D binding conformations.

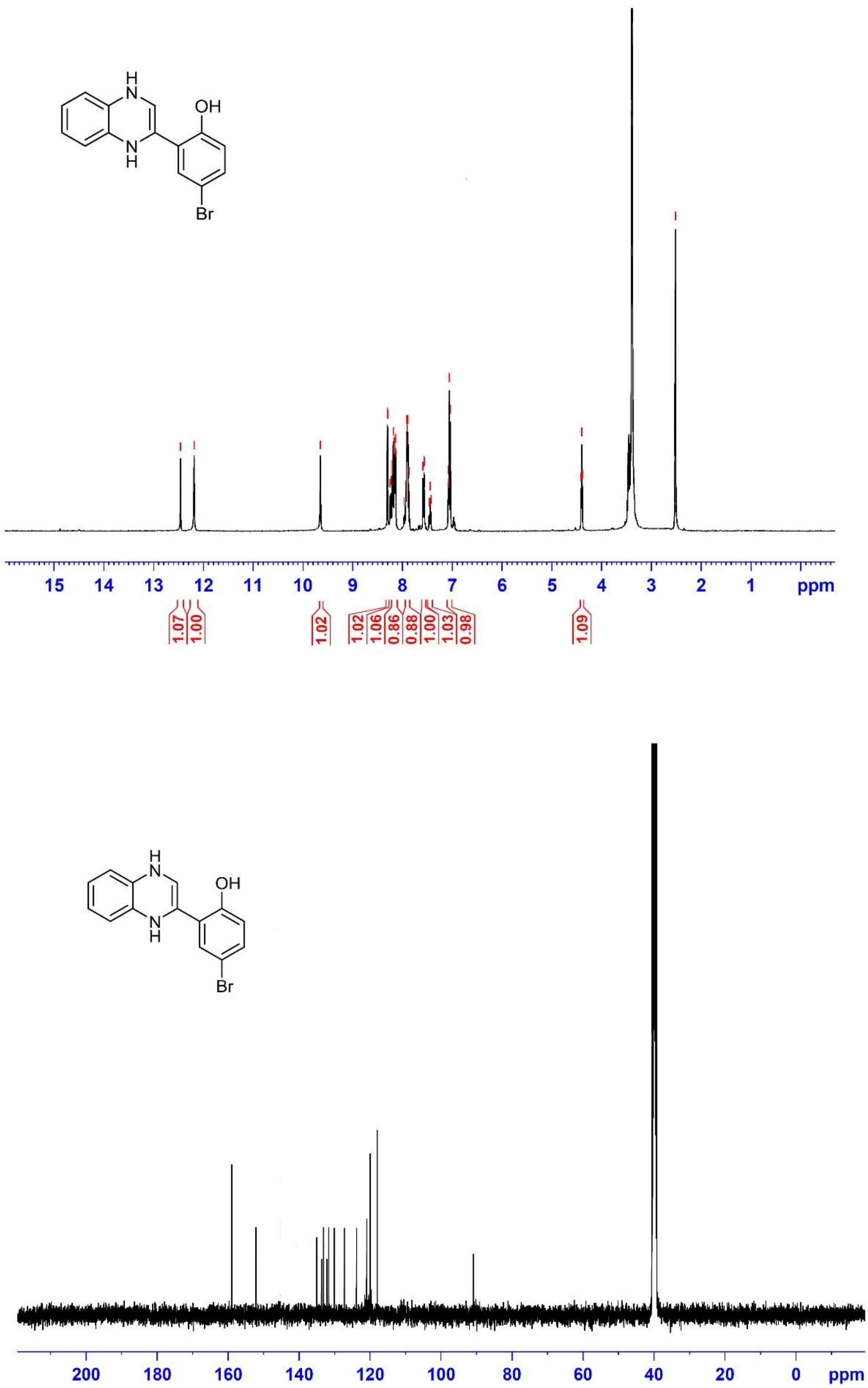

Figure S4A. ^{1}H- and ^{13}C-NMR analysis of compound **6**.

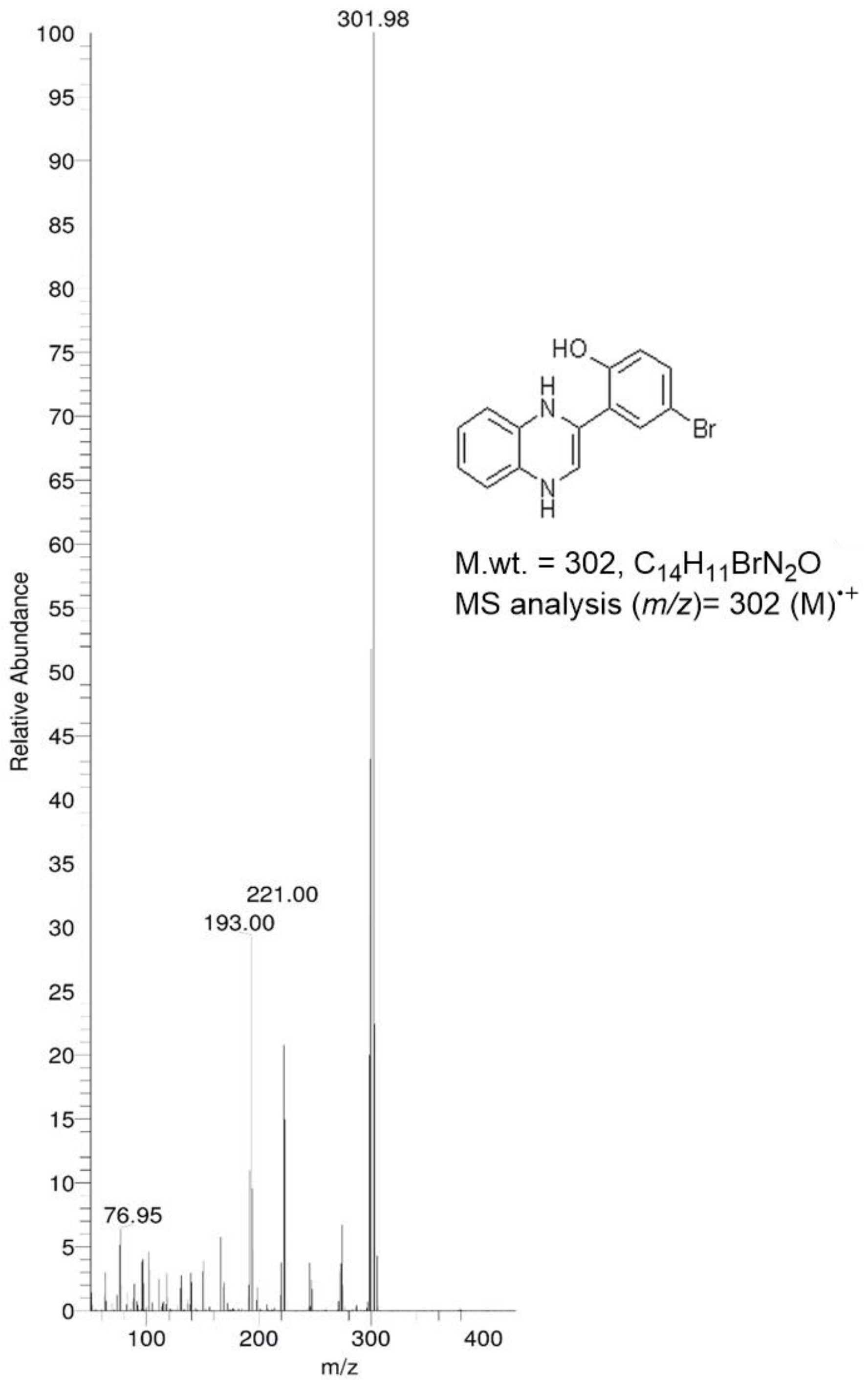

Figure S4B. Mass spectroscopic analysis of compound **6** obtained on a Finnigan MAT SSQ-7000 mass spectrometer operating under electron ionization conditions at 70 eV.

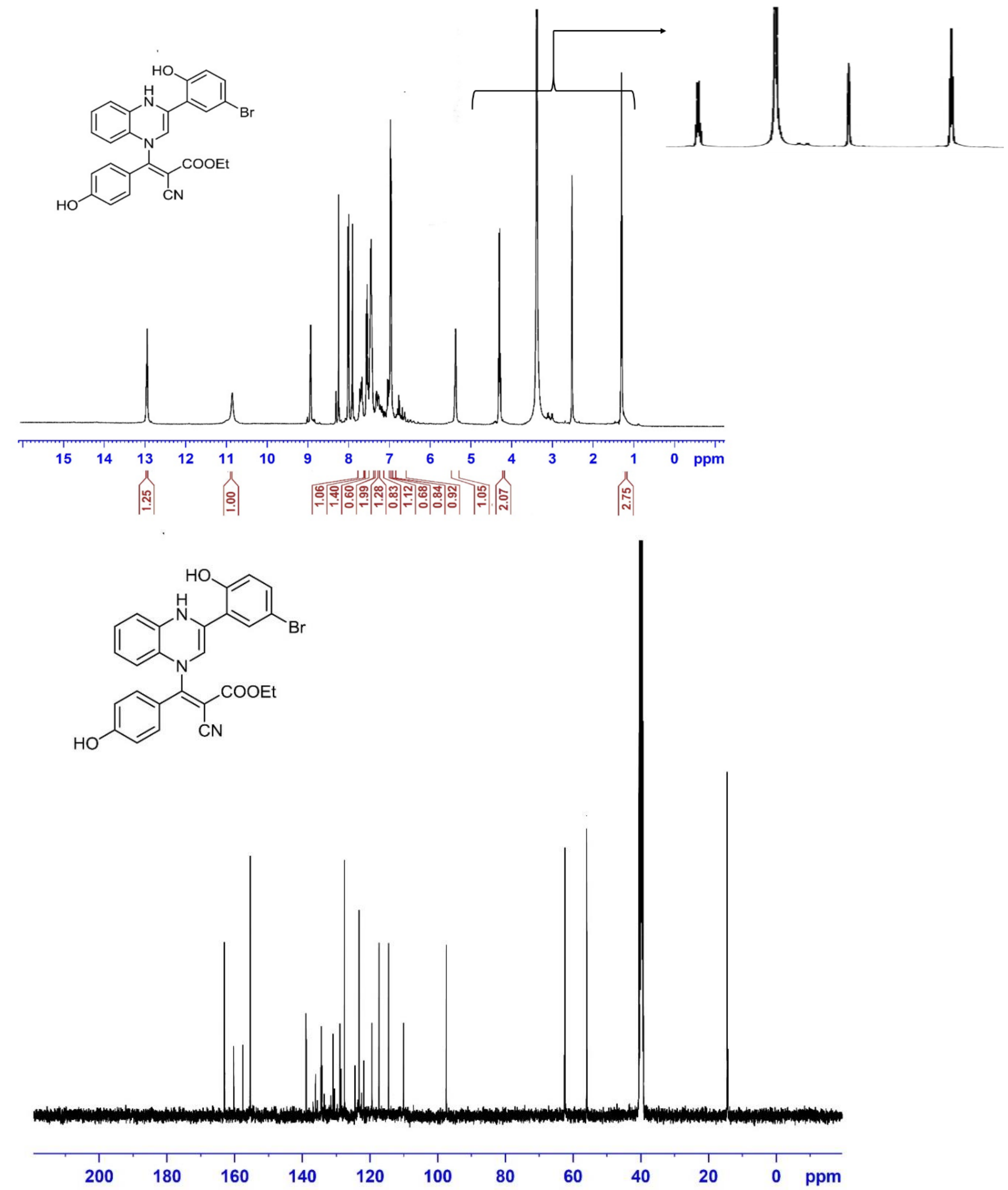

Figure S5A. ^{1}H- and ^{13}C-NMR analysis of compound **7**.

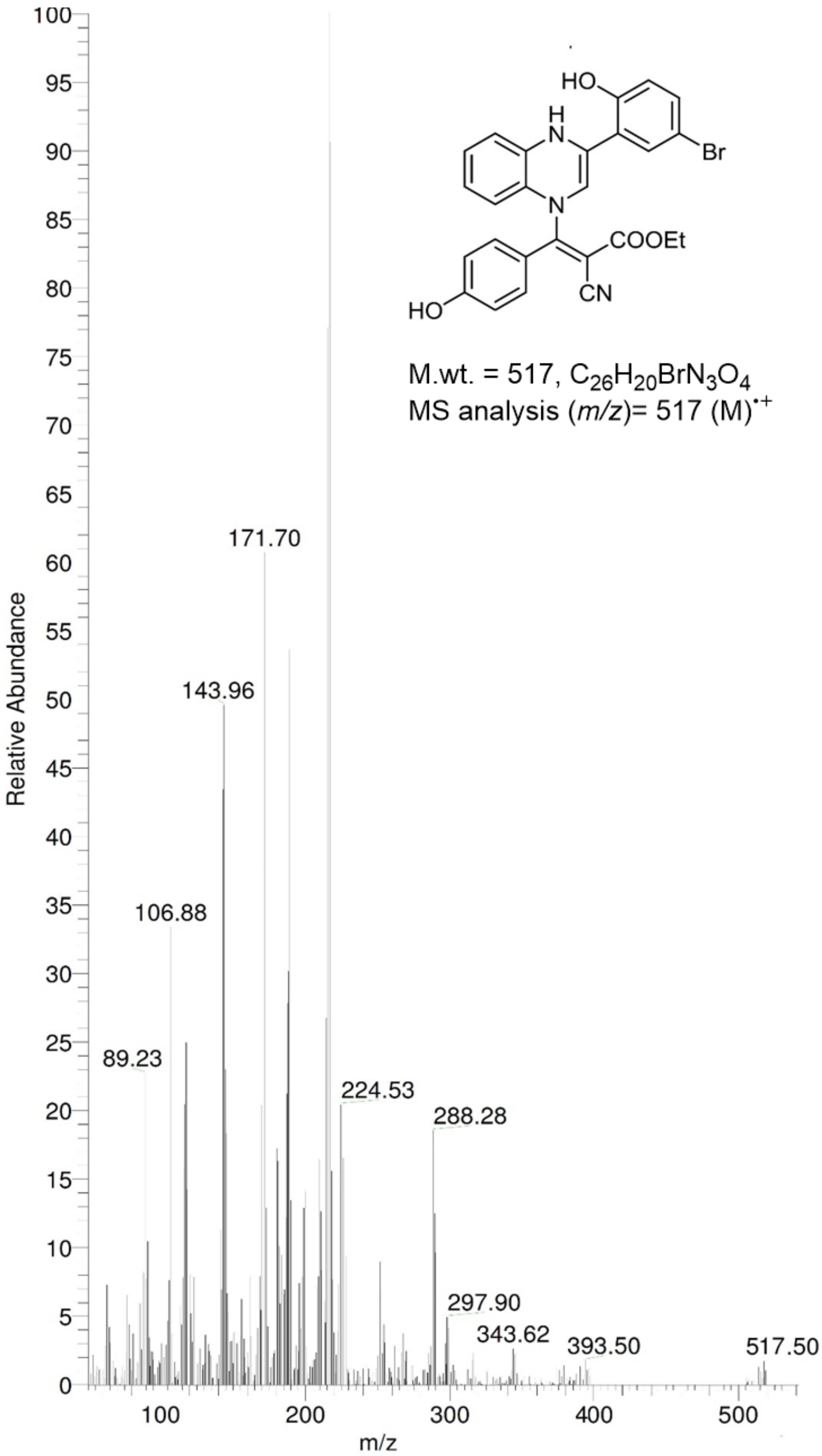

Figure S5B. Mass spectroscopic analysis of compound **7** obtained on a Finnigan MAT SSQ-7000 mass spectrometer operating under electron ionization conditions at 70 eV.

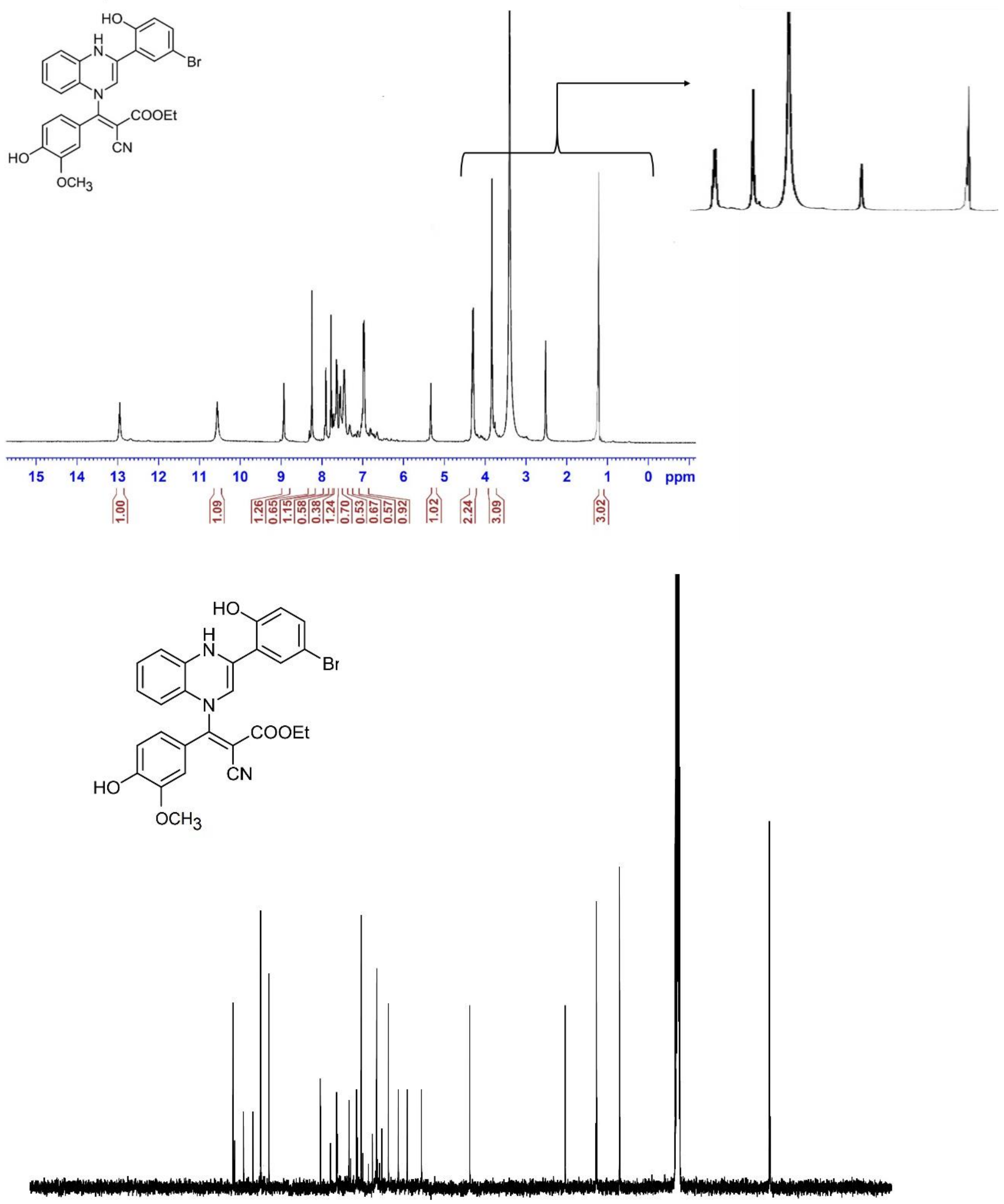

Figure S6A. ^{1}H- and ^{13}C-NMR analysis of compound **8**.

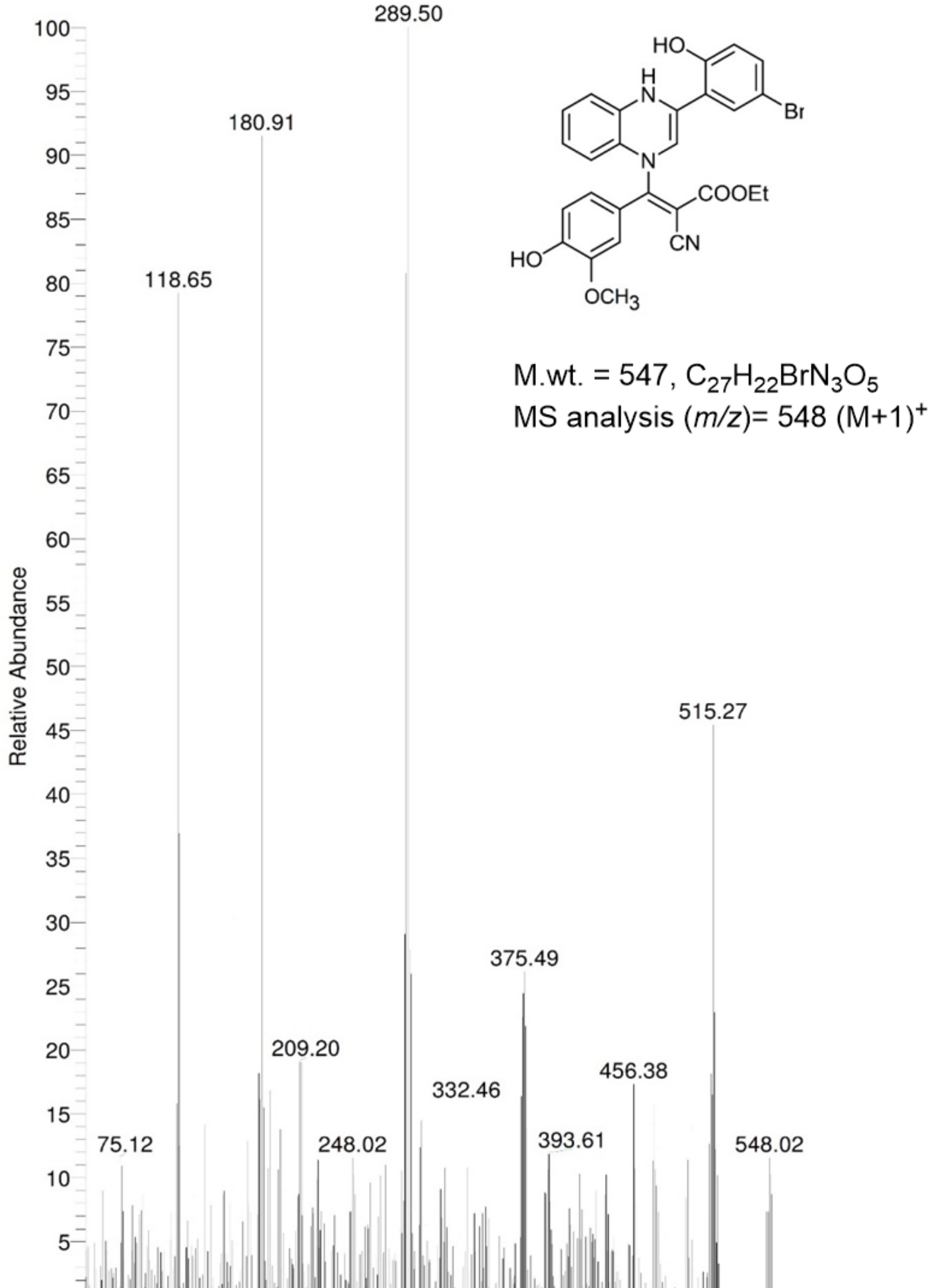

Figure S6B. Mass spectroscopic analysis of compound **8** obtained on a Finnigan MAT SSQ-7000 mass spectrometer operating under electron ionization conditions at 70 eV.

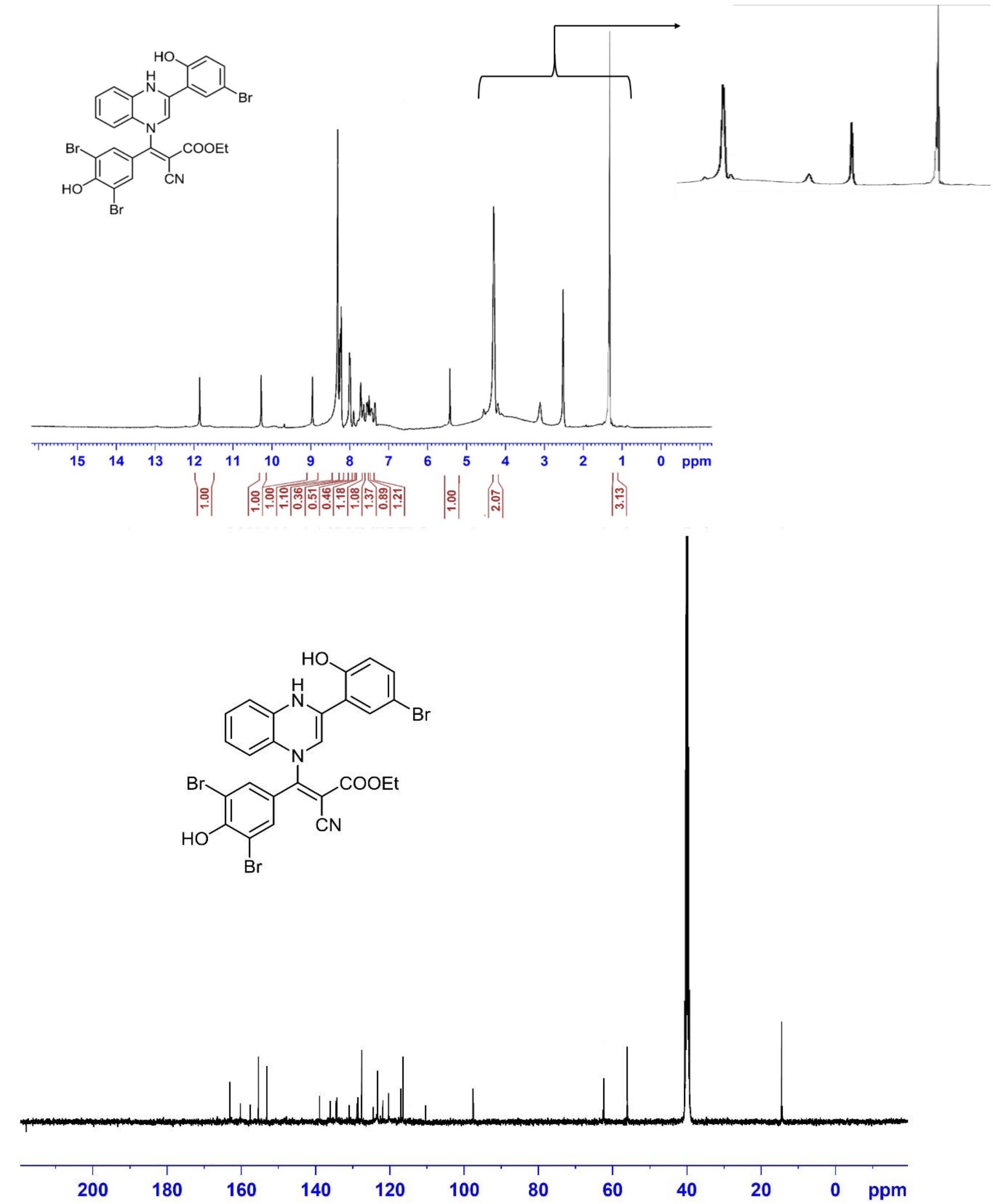

Figure S7. ^{1}H- and ^{13}C-NMR analysis of compound **9**.

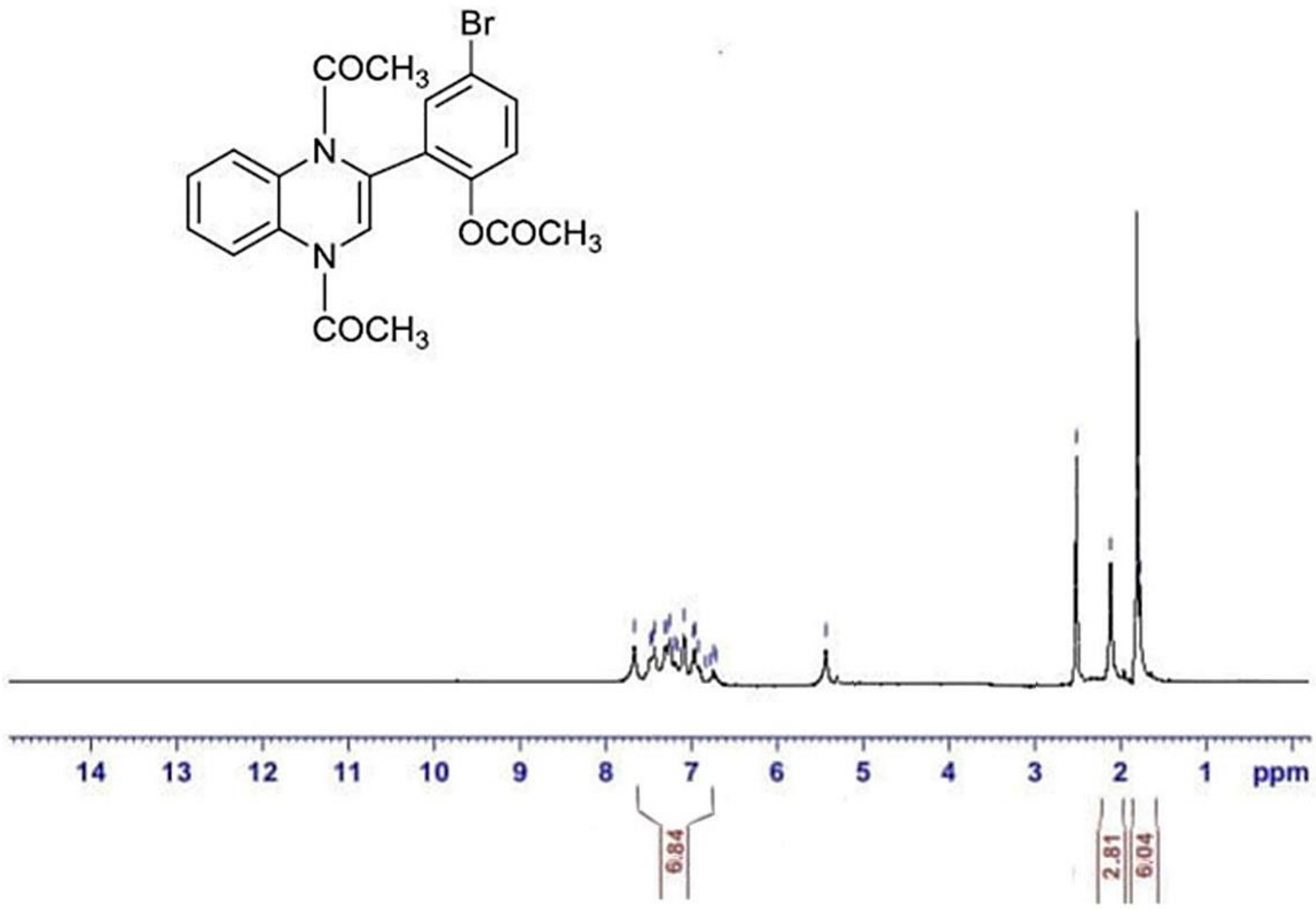

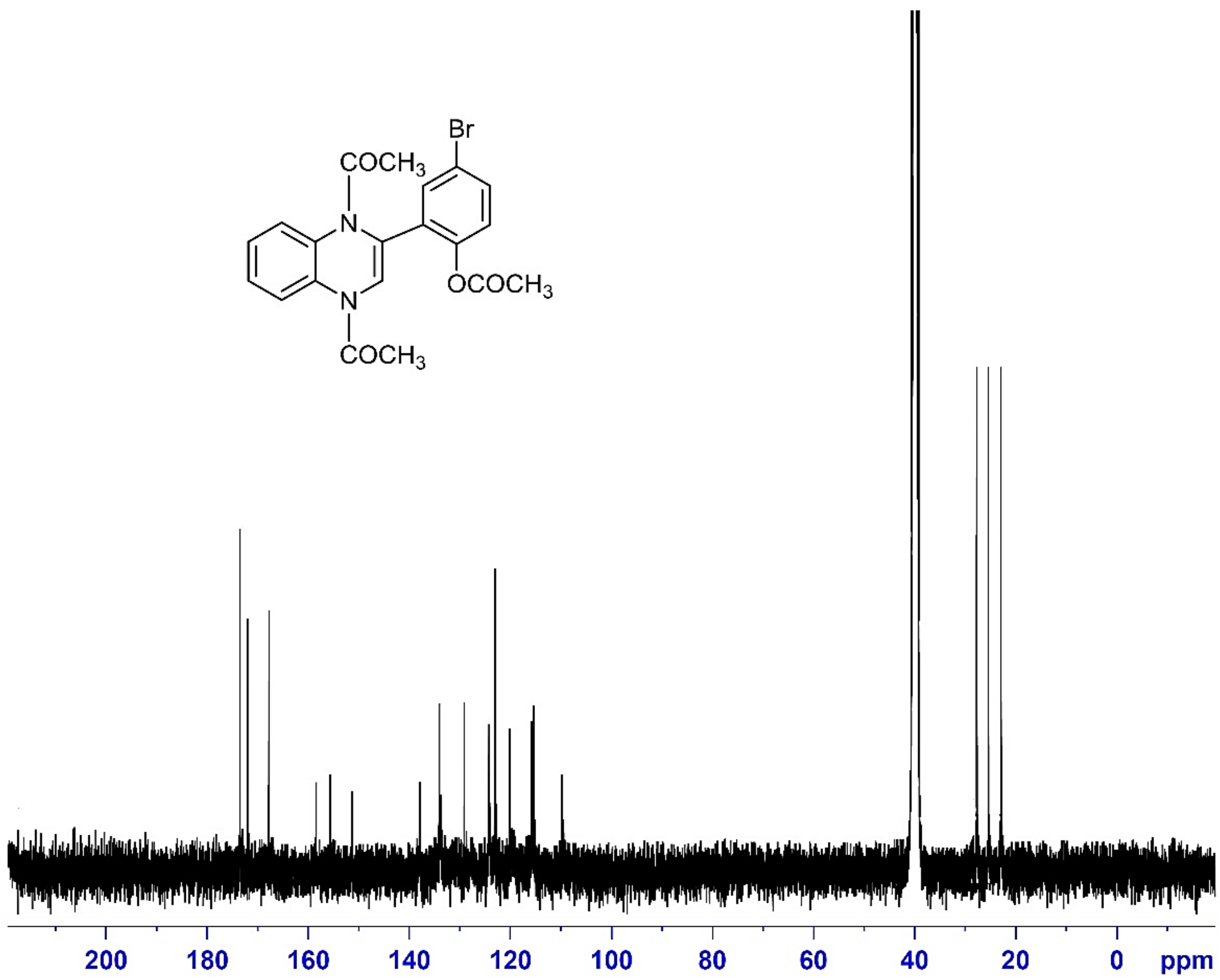

Figure S8A. ^{1}H- and ^{13}C-NMR analysis of compound **10**.

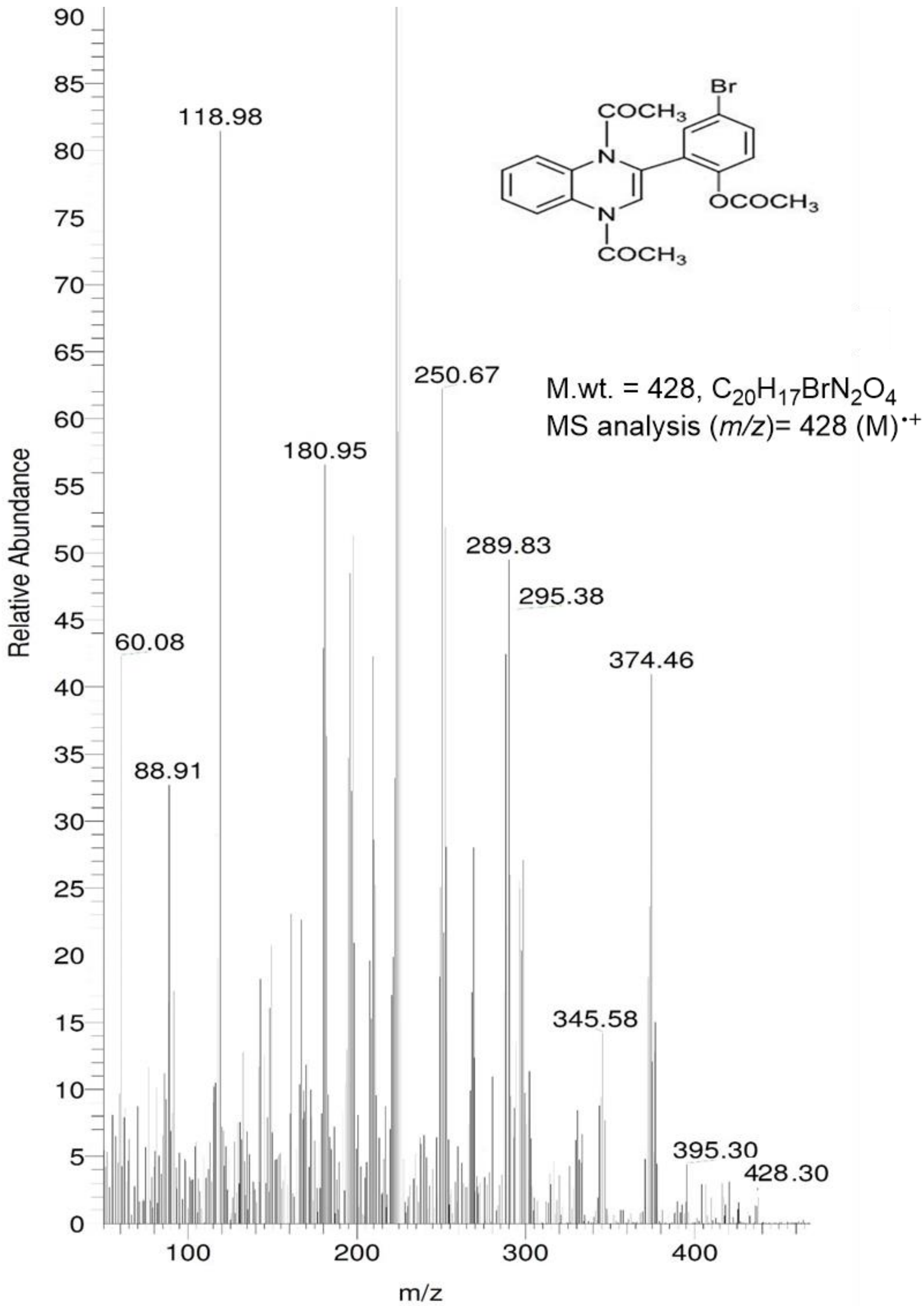

Figure S8B. Mass spectroscopic analysis of compound **10** obtained on a Finnigan MAT SSQ-7000 mass spectrometer operating under electron ionization conditions at 70 eV.

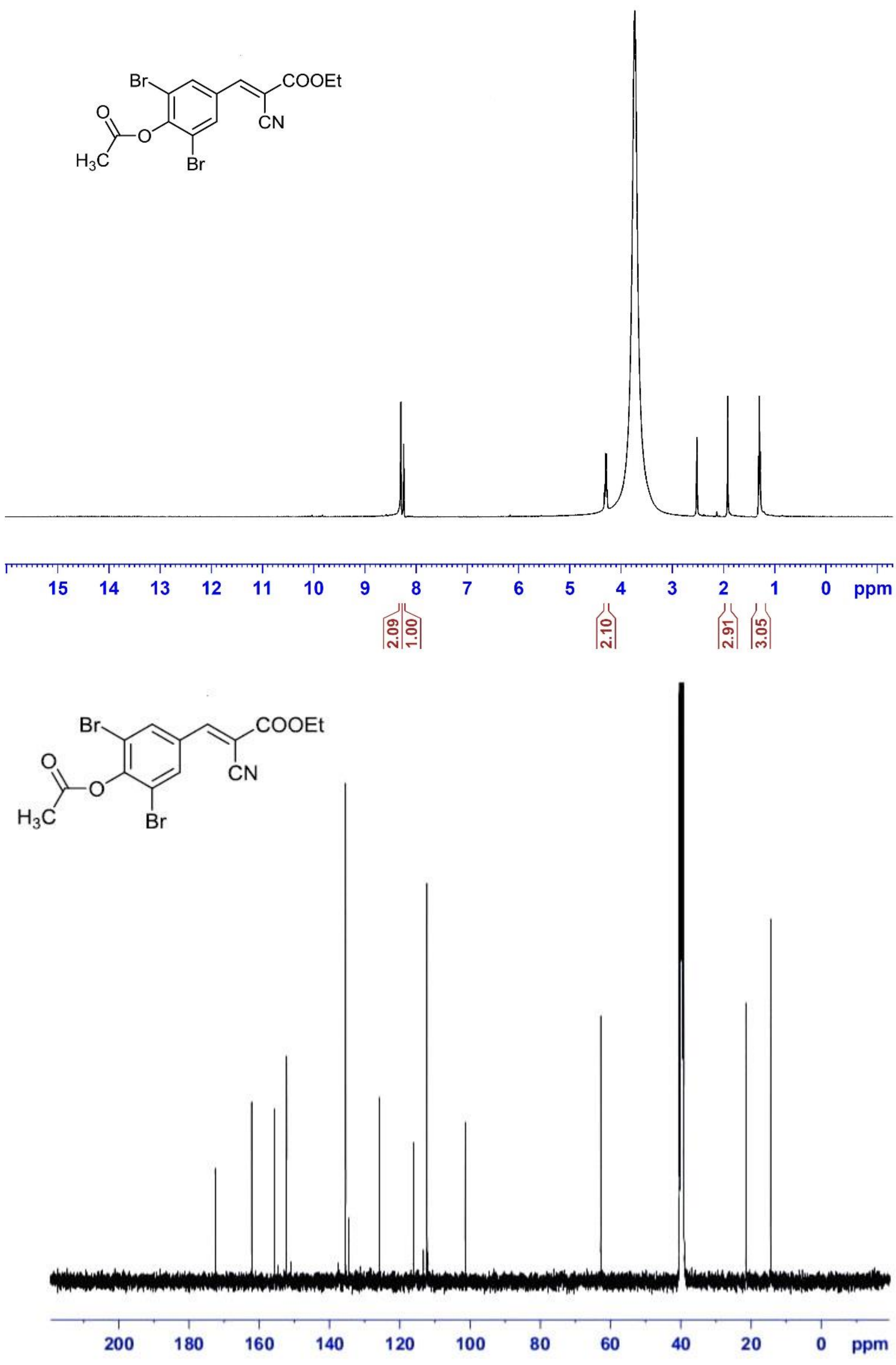

Figure S9. ^{1}H- and ^{13}C-NMR analysis of compound **11**.
